# Detection of early seeding of Richter transformation in chronic lymphocytic leukemia

**DOI:** 10.1038/s41591-022-01927-8

**Published:** 2022-08-11

**Authors:** Ferran Nadeu, Romina Royo, Ramon Massoni-Badosa, Heribert Playa-Albinyana, Beatriz Garcia-Torre, Martí Duran-Ferrer, Kevin J. Dawson, Marta Kulis, Ander Diaz-Navarro, Neus Villamor, Juan L. Melero, Vicente Chapaprieta, Ana Dueso-Barroso, Julio Delgado, Riccardo Moia, Sara Ruiz-Gil, Domenica Marchese, Ariadna Giró, Núria Verdaguer-Dot, Mónica Romo, Guillem Clot, Maria Rozman, Gerard Frigola, Alfredo Rivas-Delgado, Tycho Baumann, Miguel Alcoceba, Marcos González, Fina Climent, Pau Abrisqueta, Josep Castellví, Francesc Bosch, Marta Aymerich, Anna Enjuanes, Sílvia Ruiz-Gaspà, Armando López-Guillermo, Pedro Jares, Sílvia Beà, Salvador Capella-Gutierrez, Josep Ll. Gelpí, Núria López-Bigas, David Torrents, Peter J. Campbell, Ivo Gut, Davide Rossi, Gianluca Gaidano, Xose S. Puente, Pablo M. Garcia-Roves, Dolors Colomer, Holger Heyn, Francesco Maura, José I. Martín-Subero, Elías Campo

**Affiliations:** 1 Institut d’Investigacions Biomèdiques August Pi i Sunyer (IDIBAPS), Barcelona, Spain; 2 Centro de Investigación Biomédica en Red de Cáncer (CIBERONC), Madrid, Spain; 3 Barcelona Supercomputing Center (BSC), Barcelona, Spain; 4 CNAG-CRG, Centre for Genomic Regulation (CRG), Barcelona Institute of Science and Technology (BIST), Barcelona, Spain; 5 Wellcome Sanger Institute, Hinxton, UK; 6 Departamento de Bioquímica y Biología Molecular, Instituto Universitario de Oncología, Universidad de Oviedo, Oviedo, Spain; 7 Hospital Clínic of Barcelona, Barcelona, Spain; 8 Omniscope, Barcelona, Spain; 9 Universitat de Barcelona, Barcelona, Spain; 10 Division of Hematology, Department of Translational Medicine, University of Eastern Piedmont, Novara, Italy; 11 Biología Molecular e Histocompatibilidad, IBSAL-Hospital Universitario, Centro de Investigación del Cáncer-IBMCC (USAL-CSIC), Salamanca, Spain; 12 Hospital Universitari de Bellvitge-Institut d’Investigació Biomédica de Bellvitge (IDIBELL), L’Hospitalet de Llobregat, Barcelona, Spain; 13 Department of Hematology, Vall d’Hebron Institute of Oncology, Vall d’Hebron University Hospital, Barcelona, Spain; 14 Institute for Research in Biomedicine (IRB Barcelona), The Barcelona Institute of Science and Technology, Barcelona, Spain; 15 Universitat Pompeu Fabra (UPF), Barcelona, Spain; 16 Institució Catalana de Recerca i Estudis Avançats (ICREA), Barcelona, Spain; 17 Oncology Institute of Southern Switzerland, Bellinzona, Switzerland; 18 Institut d’Investigació Biomèdica de Bellvitge (IDIBELL), L’Hospitalet de Llobregat, Barcelona, Spain; 19 Myeloma Service, Sylvester Comprehensive Cancer Center, University of Miami, Miami, FL USA; 20 Present Address: Hospital Universitario 12 de Octubre, Madrid, Spain

**Keywords:** Chronic lymphocytic leukaemia, Cancer genomics, Cancer epigenetics, Tumour heterogeneity, B-cell lymphoma

## Abstract

Richter transformation (RT) is a paradigmatic evolution of chronic lymphocytic leukemia (CLL) into a very aggressive large B cell lymphoma conferring a dismal prognosis. The mechanisms driving RT remain largely unknown. We characterized the whole genome, epigenome and transcriptome, combined with single-cell DNA/RNA-sequencing analyses and functional experiments, of 19 cases of CLL developing RT. Studying 54 longitudinal samples covering up to 19 years of disease course, we uncovered minute subclones carrying genomic, immunogenetic and transcriptomic features of RT cells already at CLL diagnosis, which were dormant for up to 19 years before transformation. We also identified new driver alterations, discovered a new mutational signature (SBS-RT), recognized an oxidative phosphorylation (OXPHOS)^high^–B cell receptor (BCR)^low^-signaling transcriptional axis in RT and showed that OXPHOS inhibition reduces the proliferation of RT cells. These findings demonstrate the early seeding of subclones driving advanced stages of cancer evolution and uncover potential therapeutic targets for RT.

## Abstract (web summary)

Single-cell genomic and transcriptomic analyses of longitudinal samples of patients with Richter syndrome reveal the presence and dynamics of clones driving transformation from chronic lymphocytic leukemia years before clinical manifestation

## Main

Clonal evolution^1^ drives cancer initiation, progression and relapse due to the stepwise acquisition and/or selection of fitter subclones^2,3^. The understanding of tumor evolution is hampered by the analysis of bulk tumor cell populations at low resolution and at single or limited time points of the disease course in most studies^4^. A better knowledge of this process might translate into anticipation-based treatment strategies^5^. RT in CLL represents a paradigmatic model of cancer evolution occurring rarely in treatment-naive patients with CLL but found in 4–20% of patients after chemoimmunotherapy (CIT) and targeted therapies^6^. RT sometimes occurs within the first months after treatment initiation^7–9^, suggesting selection of pre-existing subclones^10^. Nonetheless, the genomic/epigenomic mechanisms driving RT after CIT^11–17^ or targeted agents^18–21^ are not well known. The aims of the present study were to reconstruct the evolutionary history of RT and to reveal the molecular processes underlying this transformation.

## Results

### Genomic characterization of RT

**Fig. 1:**
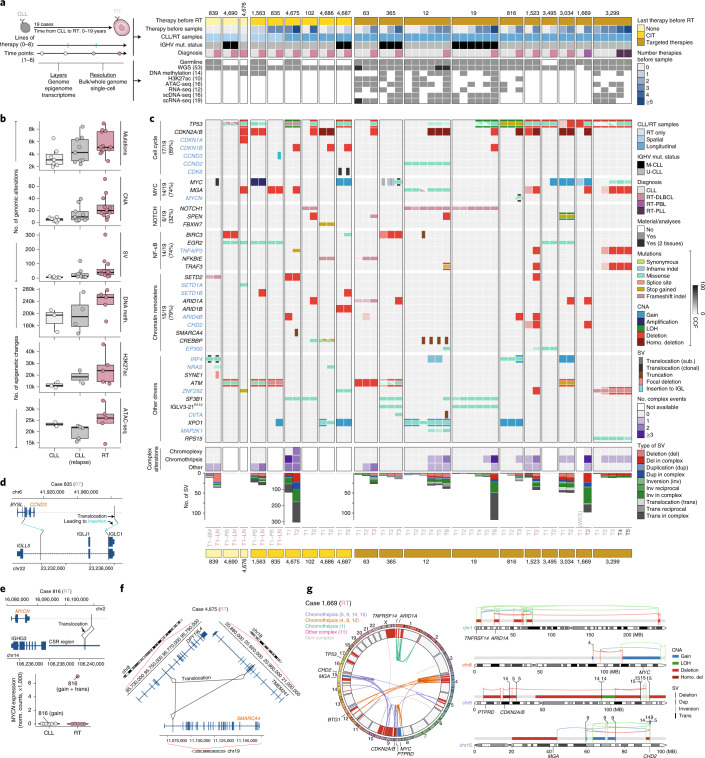
The genomic landscape of RT. **a**, Summary of the study. mut., mutation. **b**, Increase in genomic alterations and epigenetic changes compared to healthy naive and memory B cells over the disease course. Center line indicates median; box limits indicate upper and lower quartiles; whiskers indicate 1.5 × interquartile range; and points indicate individual samples. **c**, Driver alterations of CLL and RT. New drivers in RT are labeled in blue. Each column represents a sample and genes are represented in rows. The transparency of the color of mutations and CNAs indicates the cancer cell fraction (CCF). The number of tumors harboring an alteration at the time of transformation is indicated for each biological group of drivers (left). Complex structural alterations are shown below, together with the total number of SVs. LOH, loss of heterozygosity. **d**, Schema of the *CCND3* insertion next to the constant region IGLC1 in the RT sample of patient 835. **e**, Reciprocal translocation between *MYCN* and class-switch recombination (CSR) region of IGHG3 in the RT sample of patient 816 (top). *MYCN* expression based on bulk RNA-seq (bottom). **f**, Chromoplexy disrupting *SMARCA4* in the RT sample of patient 4,675. **g**, The circos plot (left) displays the SVs (links) and CNAs (inner circle) found in the RT sample of patient 1,669. CNAs are colored by type and SVs are colored according to their occurrence within specific complex events. Target driver genes are annotated. Chromosome-specific plots (right) illustrate selected complex rearrangements affecting one or multiple driver genes with CNAs and SVs colored by type.

**Extended Data Fig. 1:**
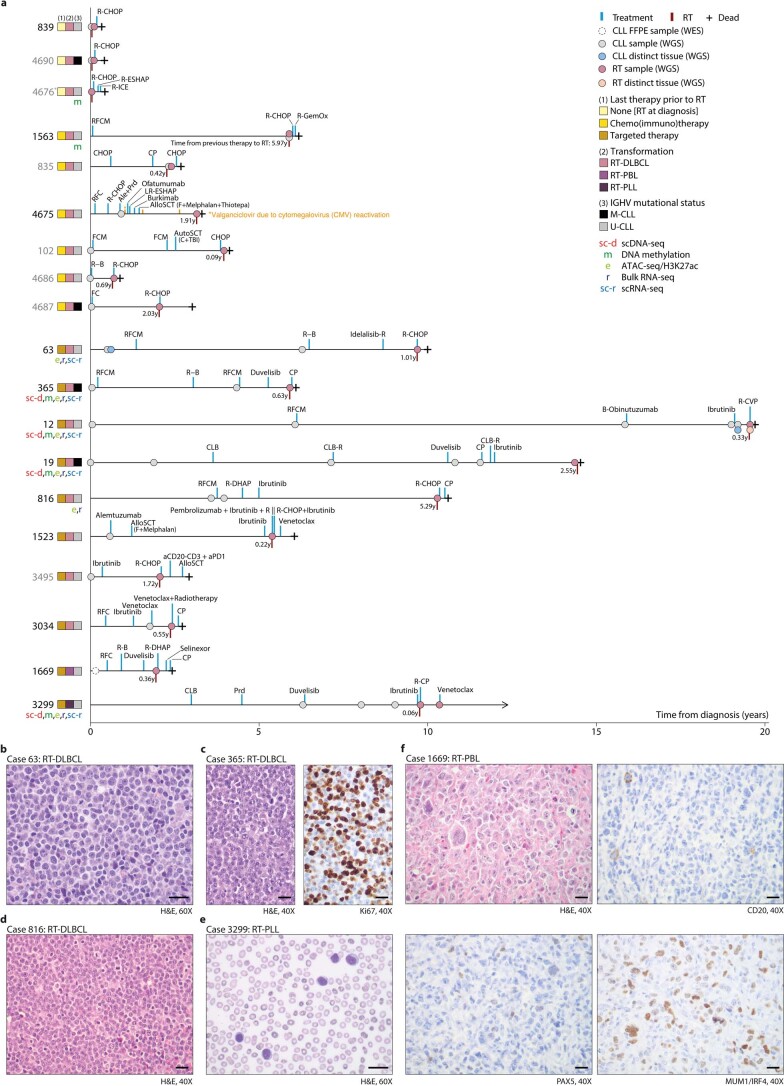
Cohort studied and types of Richter transformation. **a**. Representation of the disease course of the patients included in the study. Each sample analyzed, treatment and date of RT are depicted. Patients labeled in gray lacked germline DNA. Patient 4676 also lacked DNA from the previous CLL sample. Patients are grouped based on the last line of therapy received before RT in three groups: patients developing RT before any treatment, after chemo(immuno)therapy, and after targeted therapy. The type of transformation (RT-DLBCL, diffuse large B cell lymphoma type; RT-PLL, prolymphocytic transformation; RT-PBL, plasmablastic transformation) and IGHV mutational status are also shown. Additional molecular studies conducted in each case are also depicted. Abbreviations: Ale: alemtuzumab; AlloSCT: allogenic stem-cell transplantation; AutoSCT: autologous stem-cell transplantation; B: bendamustine; Burkimab: rituximab, methotrexate, dexametasone, ifosfamide, vincristine, etoposide, cytarabine, doxorubicin and vindesine; C: cyclophosphamide; CHOP: cyclophosphamide, doxorubicin, vincristine and prednisone; CLB: chlorambucil; CLB-R: chlorambucil and rituximab; CP: cyclophosphamide and prednisone; F: fludarabine; FCM: fludarabine, cyclophosphamide and mitoxantrone; G-GemOx: rituximab, gemcitabine, and oxaliplatin; LR-ESHAP: lenalidomide, rituximab, etoposide, methyl-prednisolone, cytarabine and cisplatin; M: mitoxantrone; Prd: prednisone; R: rituximab; R-B: rituximab and bendamustine; R-CHOP: rituximab, cyclophosphamide, doxorubicin, vincristine and prednisone; R-CVP: rituximab, cyclophosphamide, vincristine and prednisone; R-DHAP: rituximab, dexamethasone, cytarabine and cisplatin; R-ESHAP: rituximab, etoposide, methyl-prednisolone, cytarabine and cisplatin; RFC: fludarabine, cyclophosphamide and rituximab; RFCM: rituximab, fludarabine, cyclophosphamide and mitoxantrone; R-ICE: rituximab, ifosfamide, carboplatin and etoposide; TBI: total body irradiation. **b**. Morphology of the RT-DLBCL of patient 63 (hematoxylin-eosin, H&E, staining). **c**. Morphology of the RT-DLBCL of patient 365 and Ki67 staining showing high proliferative index. **d**. Morphology of the RT-DLBCL of patient 816. **e**. Morphology of the RT-PLL of patient 3299. **f**. Morphology of the RT-PBL of patient 1669 (H&E staining), which was negative for CD20 and PAX5, while positive for MUM1/IRF4. Each experiment for **b-f** was repeated twice. The scale bars in **b-f** represents 20 μm.

We sequenced 53 whole genomes and 1 whole exome of synchronous or longitudinal samples of 19 patients (up to six time points per patient) in whom CLL transformed into diffuse large B cell lymphoma (RT-DLBCL; *n* = 17), plasmablastic lymphoma (RT-PBL; *n* = 1) or prolymphocytic leukemia (RT-PLL; *n* = 1). Nontumor samples were available in 12 patients. RT occurred simultaneously with CLL at diagnosis (*n* = 3) or after up to 19 years following different lines of treatment with CIT (*n* = 6) and targeted therapies (*n* = 10; BCR inhibitors, ibrutinib *n* = 6; duvelisib *n* = 2; idelalisib *n* = 1; and BCL2 inhibitor, venetoclax *n* = 1). All instances of RT were clonally related to CLL, 15 tumors had unmutated IGHV (U-CLL) and 4 had mutated IGHV (M-CLL). Whole-genome sequencing (WGS) data were integrated with bulk epigenetic and transcriptomic analyses as well as single-cell DNA and RNA sequencing (Fig. 1a, Extended Data Fig. 1 and Supplementary Tables 1 and 2).

**Extended Data Fig. 2:**
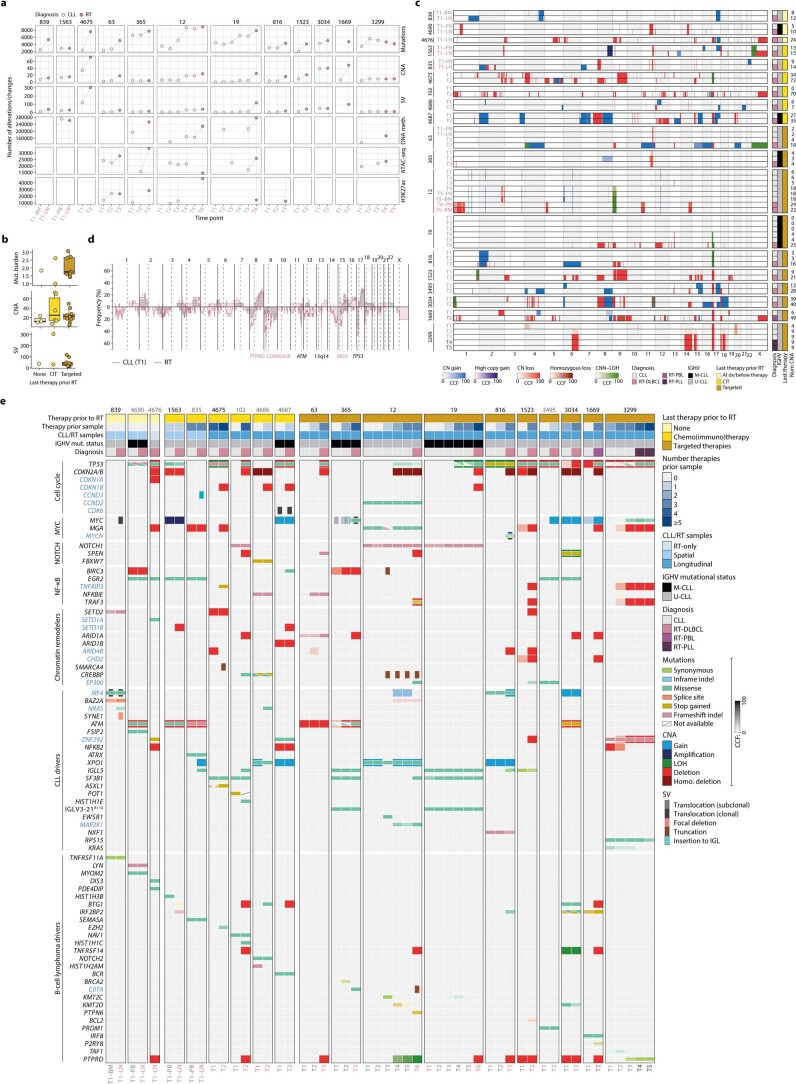
Genetic and epigenetic changes from CLL to RT, CNA profiles, and landscape of driver alterations. **a**. Number of somatic genetic alterations and epigenetic changes compared to normal counterparts along the course of the disease. Cases/time points with no grid lines correspond to unavailable data. **b**. Mutational burden, number of CNAs and number of SVs found in RT stratified according to the last therapy prior transformation. Targeted, targeted therapies. center line, median; box limits, upper/lower quartiles; whiskers, 1.5×interquartile range; points, individual samples. **c**. Copy number landscape of the studied cohort grouped by patient. The diagnosis, IGHV mutational status, last therapy prior RT, and total number of CNAs are indicated for each time point. **d**. Aggregated copy number profile of RT vs CLL. The first CLL samples (time point 1, T1) were considered. The plot shows the percentage of samples with gains (up) and losses (down). Among recurrent alterations found either in CLL or RT samples (n ≥ 5), deletions of 9p (*PTPRD* and *CDKN2A/B*) and deletions of 15q (*MGA*) were enriched in RT whereas deletions of *ATM* (11q), *TP53* (17p), and 13q14 were found at similar frequencies in CLL and RT. **e**. Oncoprint of putative driver alterations. Samples, grouped by patient (patient id at the top), are represented by columns while genes in rows. Novel drivers in RT are labeled in blue. Genes are grouped according to their biological function or if they were previously described as potential driver genes in CLL and/or mature B cell lymphomas. Metadata including the type of therapy before RT, number of treatment lines before each sample, the spatial/longitudinal nature of the CLL/RT samples analyzed, IGHV mutational status, and diagnosis is detailed in the upper rows. In the main plot, mutations (SNVs and indels) are depicted with horizontal rectangles, CNAs using the background color of each cell, and SVs with vertical rectangles. The transparency of the color of mutations and CNAs indicates the cancer cell fraction (CCF). For patients lacking the germline sample (patient id indicated in gray), the CCF of the alterations could not be inferred and a CCF of 100% was used for illustrative purposes.

**Extended Data Fig. 3:**
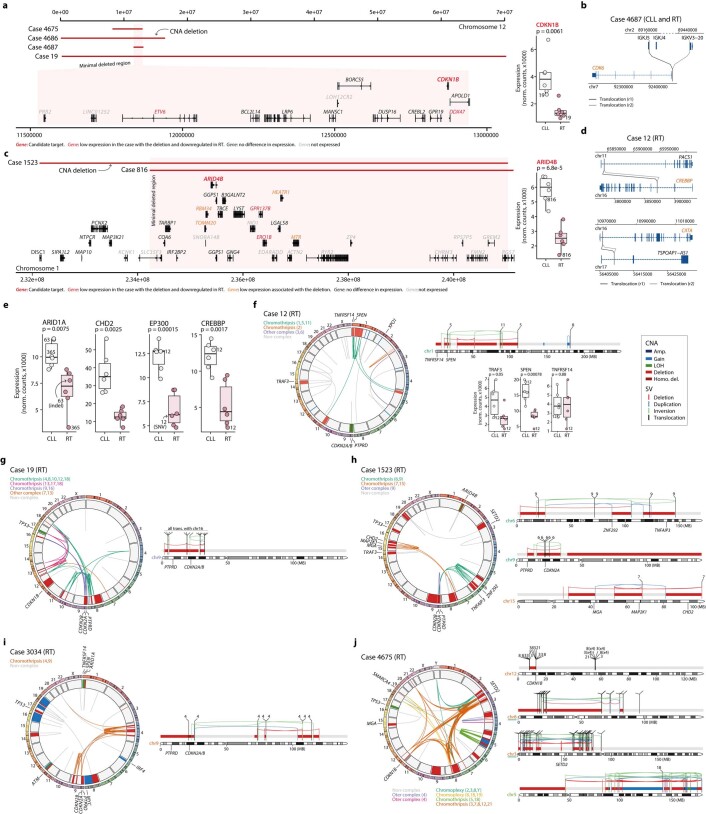
Complex genomic rearrangements affecting driver genes. **a**. Deletions in chr12 identified in four cases with the minimal deleted region affecting *CDKN1B*, which expression in CLL and RT sample pairs is shown on the right. The case carrying the deletion at time of RT is labeled in the boxplot. **b**. Reciprocal translocation juxtaposing *CDK6* next to IGKJ5 in patient 4687. **c**. Deletion in chr1 affecting two cases with the minimal deleted region targeting *ARID4B*. Its expression in CLL and RT sample pairs is shown in the boxplot on the right. **d**. Reciprocal translocations truncating *CREBBP* and *CIITA* in the RT sample of patient 12. **e**. Expression levels of known and novel RT-driver genes in CLL and RT paired samples. Cases carrying deletions/mutations at time of RT are labeled. **f-j**. Complex genomic rearrangements affecting driver genes in five selected RT samples. The circos plots show the SVs (inner links) and CNAs (middle circle) found in each sample. SVs are colored based on whether they are part of a complex event, while CNAs are painted according to their type. Chromosome-specific plots on the right show the main chromosomes affected by complex events targeting driver genes (annotated at the bottom). In these chromosome-specific plots, the color of both CNAs and SVs indicates their type. For patient 12 (f), the expression levels of three genes affected by simple (*TRAF3*) and complex (*SPEN* and *TNFRS14*) chromosomal alterations are shown. For patient 4675 (j), the partner of the translocations found in chr3 and chr8 are not specified for simplicity due to the high number of clustered structural events. All boxplots: center line, median; box limits, upper/lower quartiles; whiskers, 1.5×interquartile range; points, individual samples. All p values are from two-sided T tests.

The WGS and epigenome of CLL and RT revealed a concordant increased complexity from CLL diagnosis to relapse and RT (Fig. 1b, Extended Data Fig. 2a and Supplementary Tables 3–8). The RT genomes carried a median of 1.8 mutations per megabase, 18 copy number alterations (CNAs) and 37 structural variants (SVs) that contrasted with 1.1 mutations per megabase, 4 CNAs and 5 SVs observed at CLL diagnosis. No major differences were seen among RT occurring after different therapies (Fig. 1b and Extended Data Fig. 2b). We discovered new driver genes and mechanisms in RT, expanding previous observations^12–18,21–24^ (Fig. 1c, Extended Data Fig. 2c–e, Supplementary Fig. 1 and Supplementary Tables 9 and 10). The main alterations involved cell-cycle regulators (17 of 19, 89%), chromatin modifiers (79%), MYC (74%), nuclear factor (NF)-κB (74%) and NOTCH (32%) pathways. These aberrations were simultaneously present in most cases but alterations in MYC and NOTCH pathways only co-occurred in 2 of 19 cases (Fig. 1c). Aberrations in genes such as *TP53*, *NOTCH1*, *BIRC3*, *EGR2* and *NFKBIE* were usually present and clonally dominant after the first CLL sample, whereas others were only detected at RT or during the disease course (for example *CDKN2A/B*, *CDKN1A/B*, *ARID1A*, *CREBBP*, *TRAF3* and *TNFAIP3*) (Fig. 1c). New alterations included deletions of *CDKN1A* and *CDKN1B* in five cases of RT associated with downregulation of their expression, one immunoglobulin (IG)-*CDK6* translocation and one *CCND2* mutation already present at CLL diagnosis, and *CCND3*-IG and *MYCN*-IG translocations acquired at RT in two different cases (Fig. 1d,e, Extended Data Fig. 3a,b and Supplementary Table 11). Most chromatin remodelers were affected by deletions with reduced gene expression. New alterations in this group were deletions of *ARID4B* and truncations of *CREBBP*^25^ and *SMARCA4* (ref. ^16^) by translocations and chromoplexy (Fig. 1f and Extended Data Fig. 3c–e). We also identified recurrent *IRF4* alterations in RT, which have been linked to increased MYC levels in CLL^26^. *BTK*/*PLCG2* or *BCL2* mutations were not detected in any RT after treatment with BCR or BCL2 inhibitors, respectively. Notably, the two cases of M-CLL developing RT after targeted therapies carried the IGLV3–21^R110^ mutation, which triggers cell-autonomous BCR signaling^27^ (Fig. 1c).

In addition to the high frequency of CNAs previously identified in RT^11,14^, we observed a high number of complex structural alterations (Fig. 1c). Chromothripsis was found in eight RT tumors targeting *CDKN2A/B* and the new *CDKN1B* in five and one cases, respectively, and *MYC*, *MGA*, *SPEN*, *TNFAIP3* and chromatin remodeling genes in additional cases (Fig. 1g and Extended Data Fig. 3f–j).

Altogether, our analyses expand the catalog of driver genes, pathways and mechanisms involved in RT and recognize a similar distribution of these alterations in RT after different therapies, suggesting that treatment-specific pressure is not a major determinant of the driver genomic landscape of these tumors.

### New mutational processes in RT

**Fig. 2:**
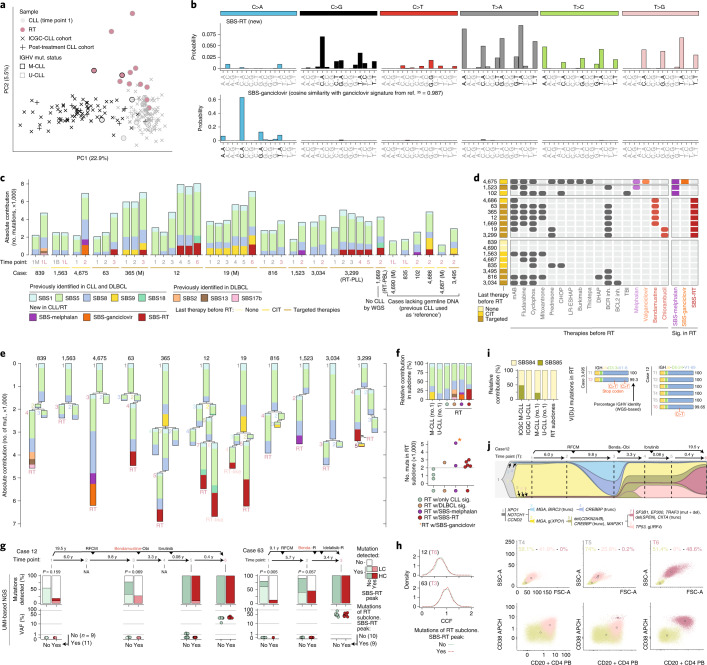
Mutational processes in RT. **a**, Principal component analysis (PCA) of the 96-mutational profile of CLL and RT. **b**, Signatures identified de novo in CLL/RT not reported in COSMIC. The main peaks of each signature are labeled in black. **c**, Contribution of mutational processes in CLL/RT. RT time points are marked in a rose color. B, peripheral blood; L, lymph node; M, bone marrow; (M), M-CLL. **d**, Therapies received before RT and presence/absence of SBS-melphalan, SBS-ganciclovir and SBS-RT at time of RT for each patient. mAB, monoclonal antibody; TBI, total body irradiation; Inh., inhibitor; Sig., signatures. **e**, Phylogenetic relationship of subclones and contribution of each mutational signature to their mutational profile. **f**, Relative contribution of mutational processes in CLL (no. 1) and RT subclones (top). Number of mutations (muts) in RT subclones (bottom). w/, with. **g**, Detection (top) and variant allele frequency (VAF) (bottom) of mutations assigned to the RT subclone during the disease course in patients 12 and 63 by high-coverage UMI-based NGS. Mutations are grouped according to the main peaks of SBS-RT. *P* values were obtained by Fisher’s test. LC, low confidence; HC, high confidence; NA, not available. **h**, Distribution of the CCF of the single-nucleotide variants (SNVs) assigned to the RT subclone based on WGS and stratified according to the main peaks of the SBS-RT. **i**, Relative contribution of mutational processes in regions of kataegis in CLL and RT (left). Two cases acquiring mutations in the immunoglobulin genes at time of RT (right). **j**, Clonal evolution along the disease course in patient 12 inferred from WGS. Abbreviations for treatment regimens are detailed in Extended Data Fig. 1a. Each subclone is depicted by a different color and number and its CCF is proportional to its height in each time point (vertical line). The phylogeny of the subclones with the main driver alterations is shown (top). Flow cytometry analysis for time points (T) 4, 5 and 6 (bottom). The size of the cells (forward scatter (FSC) versus side scatter (SSC), first row) and the expression levels of CD20 and CD38 (second row) differentiated CLL cells (yellowish) and the two larger size tumor populations (pale and dark rose color, respectively). Numbers along axes are divided by 1,000.

**Extended Data Fig. 4:**
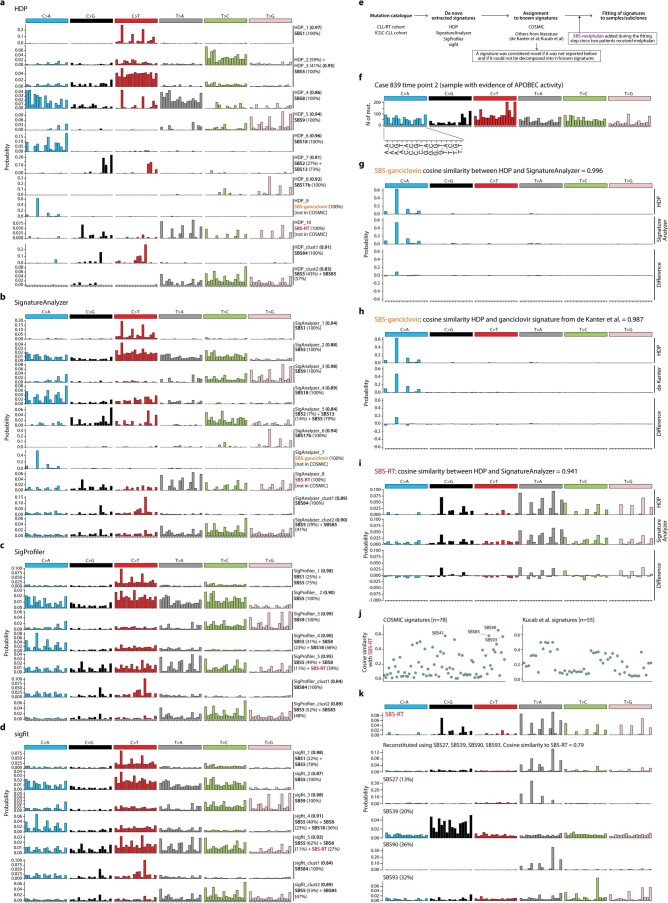
Extraction and assignment of mutational signatures. **a–d**. Signatures extracted by the Hierarchical Dirichlet Process (HDP) (a), SignatureAnalyzer (b), SigProfiler (c), and sigfit (d). COSMIC signatures needed to reconstruct the extracted signatures are shown together with their contribution (in percentage). The cosine similarities between the extracted and reconstructed signatures are shown in brackets. **e**. Workflow of the mutational signature analysis. **f**. The 96-mutation profile of the RT sample of patient 839 (time point 2), which had marked evidence of APOBEC activity (SBS2 and SBS13). **g**. Comparison of the SBS-ganciclovir extracted by HDP and SignatureAnalyzer. Based on the high cosine similarity (0.996), we considered that both signatures represented the same mutational process and selected the one extracted by HDP for downstream analyses. **h**. Comparison of the SBS-ganciclovir extracted by HDP and the ganciclovir signature reported by de Kanter et al.^35^. **i**. Comparison of the SBS-RT extracted by HDP and SignatureAnalyzer. Based on the high cosine similarity (0.941), we considered that both signatures represented the same mutational process and selected the one extracted by HDP for downstream analyses. **j**. Pairwise comparisons of the SBS-RT with known signatures from COSMIC and Kucab et al.^33^. **k**. Decomposition of the SBS-RT in “n” known signatures using an expectation maximization approach. The low cosine similarity (<0.85) between SBS-RT and the best reconstituted signature obtained using any combination of known signatures suggests that SBS-RT represents a novel mutational signature.

**Extended Data Fig. 5:**
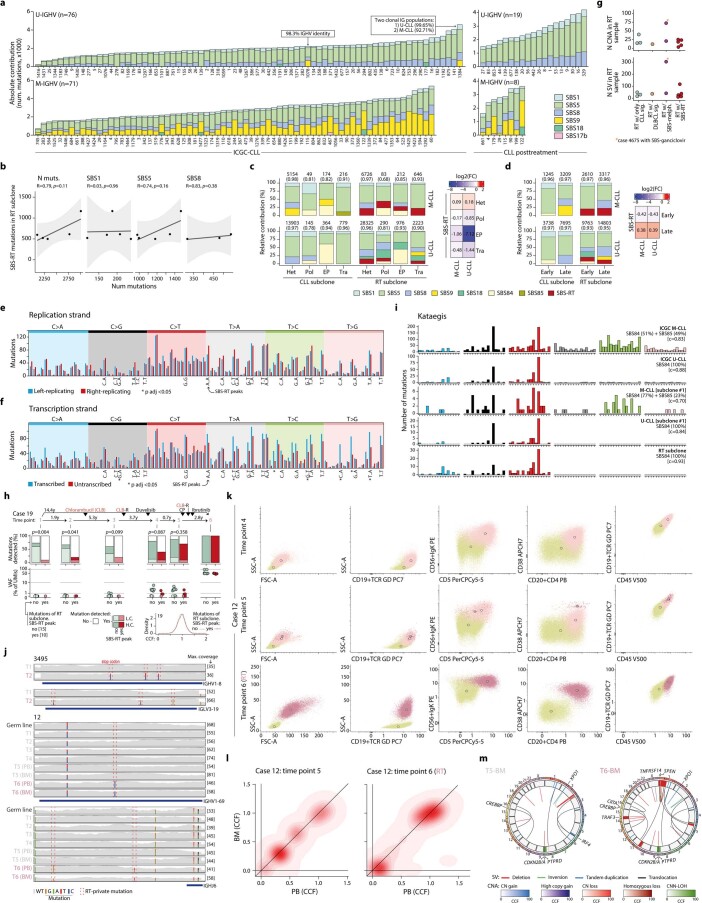
Fitting of mutational signatures, characterization of SBS-RT, and co-occurrence of RT subclones. **a**. Mutational processes in ICGC-CLL (left) and post-treatment CLL (right) cohorts. **b**. Correlation of SBS-RT with the total number of SNVs and other mutational processes in RT subclones. Gray area, 95% confidence interval. **c**. Activity of the mutational processes identified in regulatory regions of the genome: heterochromatin (Het), polycomb (Pol), enhancer/promoter (EP), and transcription (Tra). The heat map (right) shows the log2-fold change of the observed vs expected number of SBS-RT mutations/region. **d**. Contribution of the mutational processes in early/late replication regions. **e-f**. Replication (e) and transcriptional (f) strand bias of the mutational profile of RT subclones with SBS-RT. The main peaks of the SBS-RT are indicated with their context on the x-axis. Significant asymmetries are indicated with asterisks (exact *p* values are listed in Supplementary Table 16). **g**. Number of CNAs and SVs in RT samples. **h**. Detection (top) and variant allele frequency (VAF) (bottom) of mutations assigned to the RT subclone during the disease course in patient 19 based on UMI-based NGS. Mutations are grouped according to the main peaks of SBS-RT. *P* values by Fisher’s test. L.C., low confidence; H.C., high confidence. Density plot showing the distribution of the cancer cell fraction (CCF) of the SNVs assigned to the RT subclone by WGS (bottom right). **i**. Mutational profiles of kataegis in ICGC-CLL samples (row 1–2), CLL subclones from the present CLL/RT cohort (row 3–4), and RT subclones (all U-CLL) (row 5). Mutational processes identified are indicated together with its contribution and cosine similarity to the reconstructed profile. **j**. Immunoglobulin genes of two cases harboring RT-specific SNVs at time of RT (time points, T, highlighted in rose). PB, peripheral blood. BM, bone marrow. **k**. Complete flow cytometry analysis in case 12. Numbers along axes are divided by 1000. **l**. Density plot showing the comparison of the CCF of the SNVs of synchronous BM and PB samples analyzed in patient 12. **m**. Circos plots of the BM samples of patient 12 for comparison with the rearrangements observed at PB (Supplementary Fig. 1).

To understand the increased mutational burden of RT, we explored the mutational processes re-shaping the genome of CLL and RT. An unsupervised analysis showed that the mutational profile of RT was notably different from M-CLL and U-CLL before therapy (ICGC-CLL cohort, *n* = 147)^28^ or at post-treatment relapse (independent cohort of 27 CLL post-treatment samples) (Fig. 2a). We identified 11 mutational signatures distributed genome-wide and 2 in clustered mutations (Extended Data Fig. 4 and Supplementary Tables 12–14). Among the former, we extracted a new signature characterized by (T>A)A and, to a lesser extent, (T>C/G)A mutations not recognized previously in any cancer type, including CLL and DLBCL^28–33^. We named this single-base substitution signature, SBS-RT (Fig. 2b). SBS-RT was present in the RT sample of 7 of 18 patients, 1 of 6 after CIT and 6 of 10 after multiple therapies, including targeted agents and detected in all subtypes of transformation (RT-DLBCL, RT-PBL and RT-PLL) (Fig. 2c and Supplementary Table 15). It was also present in CLL samples before RT in patients 12 and 3,299 but was not identified in the reanalysis of our ICGC-CLL or post-treatment CLL cohorts. None of the patients in these two additional cohorts had evidence of RT (median follow-up 9.8 years, range 0.2–30.4) (Fig. 2c, Extended Data Fig. 5a and Supplementary Table 15). Further characterization of this new signature showed (1) a modest correlation between SBS-RT and total number of mutations (*R* = 0.79, *P* = 0.11); (2) SBS-RT mutations present in all different chromatin states and early/late replicating regions although with a moderate enrichment in heterochromatin/late replication; and (3) lack of replication and transcriptional strand bias (Extended Data Fig. 5b–f and Supplementary Table 16).

Among the remaining ten genome-wide signatures, five were previously identified in CLL and DLBCL (SBS1 and SBS5 (clock-like), SBS8 (unknown etiology), SBS9 (attributed to polymerase eta) and SBS18 (possibly damage by reactive oxygen species)); three had been only found in DLBCL (SBS2 and SBS13 (APOBEC enzymes) and SBS17b (unknown)); and two have been recently described related to treatments with melphalan^34^ or ganciclovir^35^, which were named here as SBS-melphalan and SBS-ganciclovir, respectively (Fig. 2b,c and Extended Data Fig. 4). SBS-melphalan was found in three RT cases, two had received melphalan as a conditioning of their allogenic stem-cell transplant 1.9 and 4.2 years before RT, respectively. SBS-ganciclovir was found in the RT sample of one patient that had received valganciclovir (prodrug of ganciclovir) due to cytomegalovirus reactivation (Fig. 2c,d and Extended Data Fig. 1a). Notably, all cases with the new SBS-RT at time of RT had been treated with the alkylating agents bendamustine (*n* = 5) or chlorambucil (*n* = 2) during their CLL history at a median of 2.9 years (range 0.7 to 6.8) before RT. Contrarily, RT cases lacking the SBS-RT had never received these drugs (Fig. 2c,d and Extended Data Fig. 1a).

To time the activity of each mutational process, we reconstructed the phylogenetic tree for the 11 patients with multiple synchronous (*n* = 2) or longitudinal (*n* = 9) samples and germline available and measured the contribution of each signature to the mutational profile of each subclone. The major subclone at time of transformation was named ‘RT subclone’ (Supplementary Table 17). As expected, clock-like mutational signatures were present all along the phylogeny (constantly acquired), whereas SBS9 was found only in the trunk of the two M-CLL tumors (patients 365 and 19; early events). DLBCL-related signatures, SBS-ganciclovir, SBS-melphalan and SBS-RT were found in single RT subclones in six cases while two cases carried two simultaneous subclones with SBS-RT (patients 12 and 19) (Fig. 2e). SBS-RT represented 28.6% of the mutations acquired in RT (mean 679, range 499–1,167) and it was occasionally associated with coding mutations in driver genes (*EP300* and *CIITA*) (Fig. 2f, Extended Data Fig. 5g and Supplementary Table 16). By applying a high-coverage, unique molecular identifier (UMI)-based next-generation sequencing (NGS) approach in longitudinal samples of patients 12, 19 and 63 (Supplementary Table 18), we observed that mutations of the RT subclones found in the main peaks of the SBS-RT were mainly identified in samples collected after bendamustine or chlorambucil therapy, whereas mutations not associated with SBS-RT were detected earlier during the disease course (Fig. 2g and Extended Data Fig. 5h). These results suggest a causal link between the exposure to these drugs and SBS-RT. The finding of SBS-melphalan, SBS-ganciclovir and SBS-RT in RT argues in favor of a single-cell expansion model for RT; a single cell that can carry the footprints of cancer therapies (Fig. 2h). Contrarily, the lack of SBS-RT in the 27 post-treatment CLL samples (7 patients treated with bendamustine or chlorambucil) suggests that CLL relapse might be driven by the simultaneous expansion of different subclones, hindering the detection of SBS-RT through bulk sequencing^34,36^.

RT subclones also acquired kataegis, mainly within the immunoglobulin loci, attributed to activation-induced cytidine deaminase (AID) activity (SBS84 and SBS85)^29,32^ (Fig. 2i and Extended Data Fig. 4). These kataegis led to the acquisition of mutations in the rearranged V(D)J gene in five RT cases (one after CIT and four targeted therapies) (Fig. 2i, Extended Data Fig. 5i,j and Supplementary Table 19). This canonical AID activity in RT is concordant with the acquisition of SBS9 mutations in two RT samples (4,686 (CIT) and 3,495 (targeted therapies)) and SVs mediated by aberrant class-switch recombination or somatic hypermutation in six RT (one before therapy, two CIT and three new agents), which targeted *MYC*, *MYCN*, *TRAF3* and *CCND3* (Fig. 1c and Supplementary Table 2).

SBS-RT mutations were found in CLL samples before the transformation in patient 3,299 although it was only present in the RT subclone (Fig. 2c,e). SBS-RT was also found in two different subclones in case 12 and 19. We speculated that these secondary subclones with SBS-RT (named ‘RT-like’ subclones) could correspond to the single-cell expansion of a ‘transformed’ cell that could have been missed by the routine analysis (Fig. 2e). The reanalysis of flow cytometry data available for case 12 detected two cell populations at time point (T) 4 differing in size and surface markers (likely CLL and RT-like subclones), whereas at T5 we detected an additional population of large cells (RT subclone, 0.2% cells) that expanded at T6, substituting the previous large cell population (RT-like subclone) (Fig. 2j and Extended Data Fig. 5k–m). WGS analysis showed that the RT-like and RT subclones diverged from a cell carrying a deletion of *CDKN2A/B* and truncation of *CREBBP*, each acquiring more than 2,100 specific mutations (Fig. 2e,j).

Altogether, these findings show that RT may arise simultaneously from different subclones and that such subclones can be detectable time before their final expansion and clinical manifestation. The identification of mutations in RT associated with early-in-time CLL therapies demonstrates that RT emerges from the clonal expansion of a single cell previously exposed to these therapies.

### Dormant seeds of RT at CLL diagnosis

**Fig. 3:**
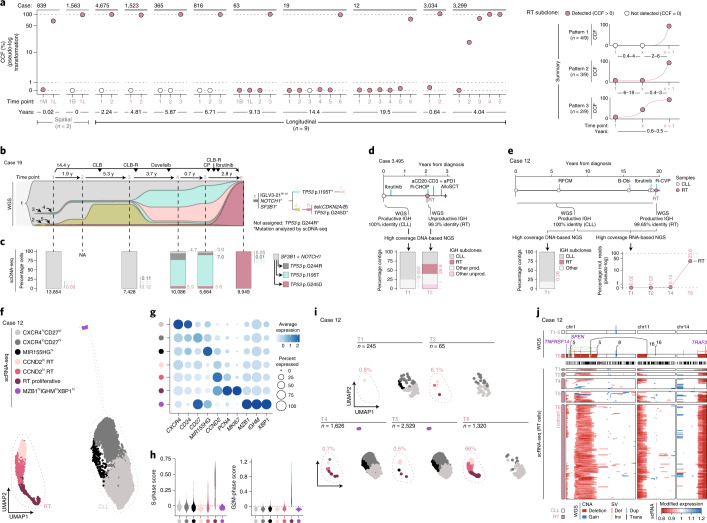
Early seeding of RT. **a**, Evolution of the RT subclone along the disease course based on WGS. Time lapse between the first and last sample analyzed (bottom). RT time points are marked in a rose color. Summary of the three patterns observed (right). **b**, Fish plot showing the clonal evolution along the course of the disease in patient 19 inferred from WGS analysis. Each subclone is depicted by a different color and number and its CCF is proportional to its height at each time point (vertical lines). Phylogeny of the subclones and main driver events (right). **c**, Mutation tree reconstructed by scDNA-seq for case 19 together with the fraction of cells carrying each specific combination of mutations in each time point. The total number of cells per sample is shown at the bottom. The number of cells assigned to each subclone is shown in Supplementary Table 20. **d**, Schematic representation of the clinical course and samples analyzed for patient 3,495 together with the size of the IGH subclones identified using high-coverage NGS analyses. Abbreviations for treatment regimens are detailed in Extended Data Fig. 1a. **e**, Clinical course and IGH subclones identified by DNA- and RNA-based NGS in patient 12. **f**, Uniform Manifold and Projection (UMAP) plot for case 12 based on the scRNA-seq data of all time points colored by annotation. **g**, Expression of key marker genes in each cluster identified in case 12. **h**, Distribution of cell-cycle phase scores for each cluster based on scRNA-seq in case 12. **i**, UMAP visualization split by time point in case 12 with the fraction of RT cells annotated. ‘*n*’, number of cells. **j**, Chromosomal alterations detected by WGS in chromosomes 1, 11 and 14 in CLL and RT samples of patient 12 (top). Copy number profile of RT cells detected at the different time points according to scRNA-seq. Only a subset of RT cells from time point 6 (time of diagnosis of RT) was included for illustrative purposes (bottom).

**Extended Data Fig. 6:**
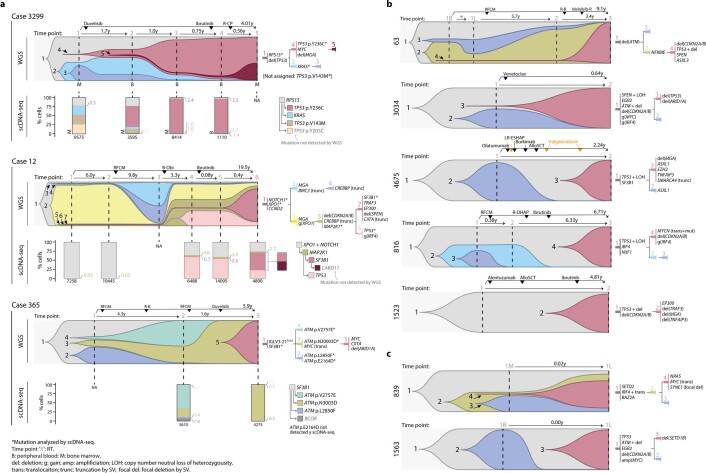
Clonal dynamics from CLL to RT. **a**. Subclonal reconstruction and clonal evolution of three cases (3299, 12 and 365) with WGS and scDNA-seq data available. The upper fish plot shows the clonal evolution along the course of the disease inferred from WGS analyses. Each color represents a different subclone and their height is proportional to their cancer cell fraction (CCF) in each time point (vertical lines). The treatments that the patient received and the elapsed time (in years) between samples are indicated at the top. The tissue is indicated for samples of patient 3299 in which different tissues were analyzed by WGS and scDNA-seq in the same time point. The phylogeny of the subclones is depicted together with the main driver alterations (top right). The lower bar plots show the dynamics of the different subclones according to the scDNA-seq analyses. The total number of cells per sample is shown at the bottom. The number of cells assigned to each subclone can be found in Supplementary Table 20. The mutation tree inferred from scDNA-seq data is shown at the bottom-right part. **b-c**. Subclonal architecture and dynamics of six cases with longitudinal samples (b) and two cases with spatial samples (c) analyzed by WGS.

The WGS-based subclonal phylogeny of the nine patients with fully characterized longitudinal samples predicted that the RT subclone was present at low cancer cell fraction (CCF) in the preceding CLL samples in five (56%) patients and only detected at time of transformation in the remaining four (44%) (Fig. 3a). Indeed, the RT subclone was detected at time of CLL diagnosis in three of five patients, remained stable at a minute size (<1%) for 6–19 years of natural and treatment-influenced CLL course and expanded at the moment of clinical manifestations (patients 12, 19 and 63) (Fig. 3a). In the other two patients, the RT subclone was also detected in the first CLL sample analyzed but rapidly expanded driving the RT 0.6 and 3.5 years later in patients 3,034 and 3,299 (RT-PLL), respectively (Fig. 3a and Extended Data Fig. 6).

We next performed single-cell DNA sequencing (scDNA-seq) of 32 genes in 16 longitudinal samples of 4 patients (12, 19, 365 and 3,299) to validate these evolutionary histories of RT (202,210 cells passing filters, mean of 12,638 cells per sample; Fig. 1a, Supplementary Fig. 2 and Supplementary Table 20). Focusing on patient 19 with a time lapse of 14.4 years from diagnosis to RT (Fig. 3b), the RT subclone (subclone 5) at transformation (T6) carried *CDKN2A/B* and *TP53* (p.G245D) alterations, whereas the main CLL subclones driving the relapse after therapy at T4 and T5 harbored a different *TP53* mutation (p.I195T; subclones 3 and 4). The WGS predicted the presence of all these subclones at CLL diagnosis (T1). Using scDNA-seq we identified two small populations accounting for 0.1% of cells carrying the *TP53* p.I195T and p.G245D mutations, respectively, at T1, which were also detected at relapse 7.2 years later (T3). The subclone carrying *TP53* p.I195T expanded to dominate the second relapse after 3.7 years at T4 and T5 but was substituted by the subclone carrying *TP53* p.G245D at T6 in the RT 14.4 years after diagnosis. All these subclones carried the *SF3B1* and *NOTCH1* mutations of the initial CLL subclone (Fig. 3c and Supplementary Table 20). The scDNA-seq of the three additional cases also corroborated the phylogenies and most of the dynamics inferred from WGS (Extended Data Fig. 6a). These results suggest that CLL evolution to RT is characterized by an early driver diversification probably generated before diagnosis, consistent with the early immunogenetic and DNA methylation diversification previously reported in CLL^37–39^ and that RT may emerge by a selection of pre-existing subclones carrying potent driver mutations rather than a de novo acquisition of leading clones.

As we identified five cases of RT carrying specific mutations in the immunoglobulin genes by WGS (Fig. 2i), we analyzed whether these immunoglobulin-based RT subclones were already present at CLL diagnosis using high-coverage NGS in patients 12 and 3,495 (Supplementary Table 21). Focusing on patient 3,495, for which the lack of germline material precluded our phylogenetic analyses, the RT occurring after treatment with ibrutinib harbored two new V(D)J mutations generating an unproductive IGH gene. NGS identified 0.002% sequences carrying the same two mutations at CLL diagnosis 1.72 years before (Fig. 3d). We also observed the expansion of additional unproductive subclones accounting for 11.8% of all sequences at time of RT, suggesting that BCR-independent subclones may have a proliferative advantage under therapy with BCR inhibitors (Fig. 3d). Similar results were found in patient 12 in which the V(D)J sequence of RT carrying a new mutation was already identified at CLL diagnosis 19.5 years before at DNA and RNA level (Fig. 3e). As the immunogenetic features represent a faithful imprint of the B cell of origin, the early identification of the same immunogenetic subclone provides further evidence for an early seeding of RT.

**Extended Data Fig. 7:**
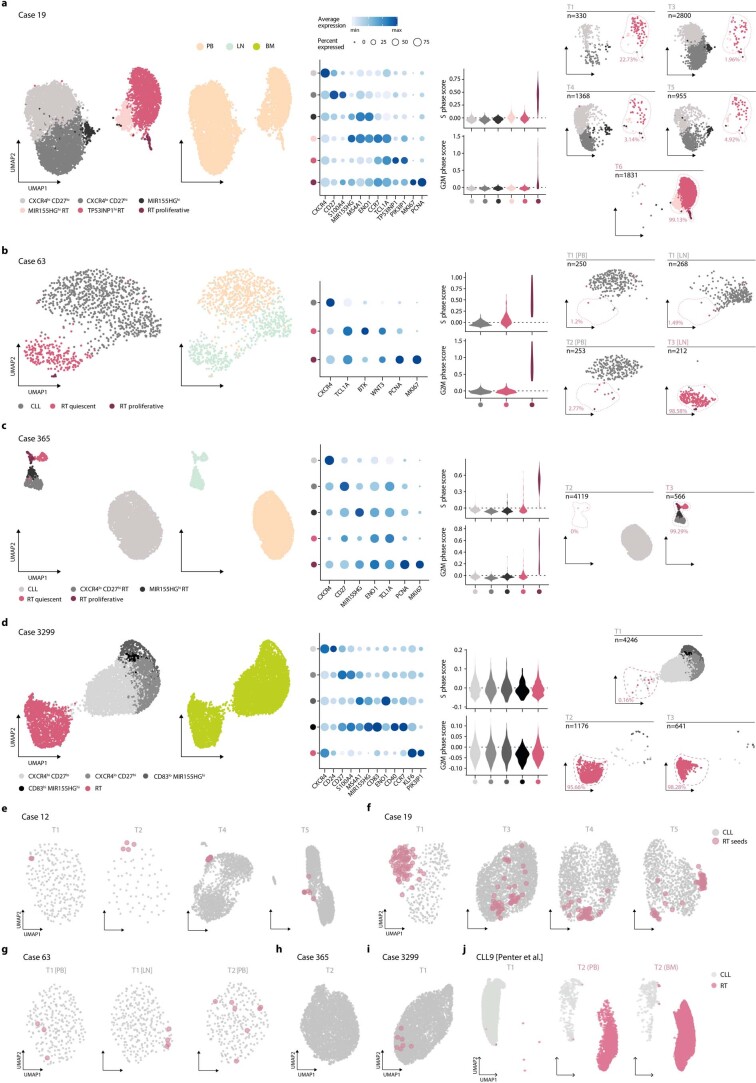
scRNA-seq characterization of CLL and RT. **a–d**. UMAP visualization of tumor cells from all time points colored by annotation and tissue of origin. hi, high; lo, low; PB, peripheral blood; LN, lymph node; BM, bone marrow (left). Dot plot with the expression of key markers in each cluster. Color and size represent scaled mean expression and proportion of cells expressing each marker gene, respectively (middle-left). Violin plots showing the cell-cycle phase scores (S and G-to-M) for each cluster of cells (middle-right). UMAP visualization split by time point (right). ‘n’ refers to the total number of cells in that time point, and the percentage refers to the proportion of cells within RT clusters. **e-i**. Time point-specific UMAP visualizations for each case. RT seed cells are depicted in rose and with an increased size. **j**. UMAP visualization of case CLL9 from Penter et al.^43^ split by time point. PB, peripheral blood; BM, bone marrow.

**Extended Data Fig. 8:**
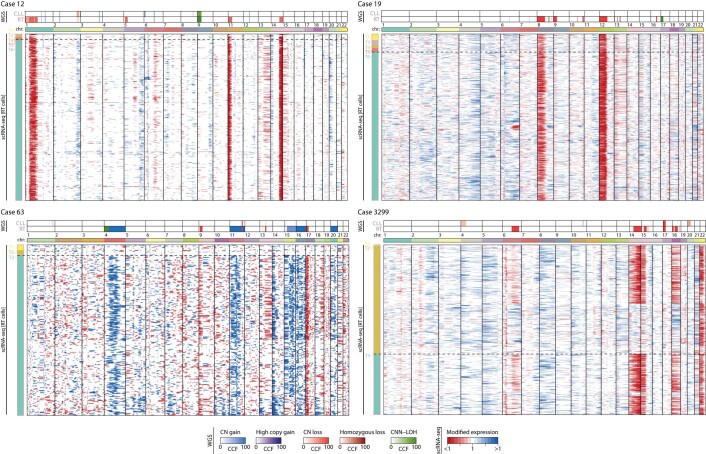
CNA profile of RT cells by scRNA-seq. For each patient, the CNA profile of CLL and RT samples according to WGS is shown (top) together with the CNA profile of each individual RT cell based on scRNA-seq (bottom). For scRNA-seq, each row represents a RT cell and the horizontal dashed line separates the RT cells identified in the time points previous to the diagnosis of RT (that is, seed RT cells) from those present in the sample collected at time of diagnosis of RT. Note that CLL cells were used as reference for CNA analyses using scRNA-seq data.

We finally tracked RT subclones during the disease course using single-cell RNA sequencing (scRNA-seq) of 19 longitudinal samples of five patients (24,800 tumor cells passing filters, mean of 1,305 cells per sample; Fig. 1a and Supplementary Table 22). As expected, RT and CLL cells had remarkably different gene expression profiles (Fig. 3f and Extended Data Fig. 7a–d). The transcriptome of CLL cells was dominated by three main clusters identified across patients and characterized by different expression of *CXCR4*, *CD27* and *MIR155HG*, respectively, which may represent the recirculation of CLL cells between peripheral blood and lymph nodes^40–42^ (Fig. 3f,g and Extended Data Fig. 7a–d). Contrarily, RT intraclonal heterogeneity was mainly related to distinct proliferative capacities with a cluster of cells showing high *MKI67* and *PCNA* expression as well as high S and G2M cell-cycle phase scores. The remaining RT clusters were characterized by the expression of different marker genes among patients, including *CCND2*, *MIR155HG* and *TP53INP1* (Fig. 3f–h and Extended Data Fig. 7a–d). When considering each time point separately, we detected RT cells in all CLL samples before transformation in patient 12, 19, 63 and 3,299 but not in patient 365 (Fig. 3i and Extended Data Fig. 7a–i). The presence and dynamics of these RT subclones according to their transcriptomic profile recapitulated the findings obtained by WGS, scDNA-seq and immunoglobulin analyses in all five patients, suggesting that they captured the same cells. Indeed, using scRNA-seq we could identify the CNAs involved in simple and complex structural alterations found at time of RT by WGS already in the dormant RT cells at CLL diagnosis and subsequent time points before their final expansion (Fig. 3j and Extended Data Fig. 8). These findings suggest an early acquisition of SVs, including chromothripsis and transcriptomic identity in RT.

To validate our observations, we reanalyzed the longitudinal scRNA-seq dataset from Penter et al.^43^ consisting of nine patients with CLL, one of which developed RT. In this case, we identified RT cells in the CLL sample collected 1.6 years before the RT (Extended Data Fig. 7j). Overall, our integrative analyses uncovered a widespread early seeding of RT cells up to 19 years before their expansion and clinical manifestation.

### OXPHOS^high^–BCR^low^ transcriptional axis of RT

**Fig. 4:**
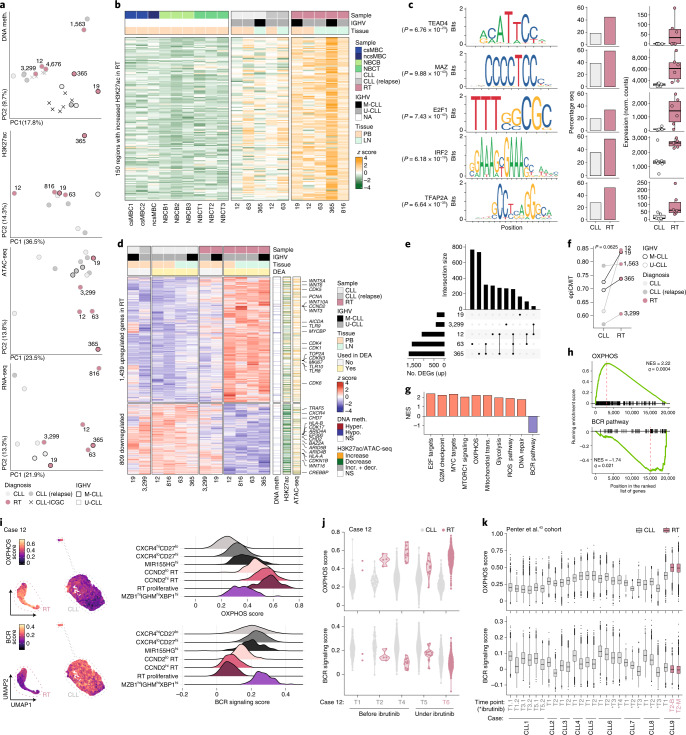
Proliferation, OXPHOS and BCR pathways dominate the epigenome and transcriptome of RT. **a**, PCA of the bulk epigenetic and transcriptomic layers analyzed. **b**, Heat map showing 150 regions with increased H3K27ac levels in RT. **c**, TF enriched within the ATAC peaks identified in the regions of increase H3K27ac in RT. The motif, percentage of RT-specific active regions and regions with increased H3K27ac in CLL that contained the motif and TF expression (bulk RNA-seq) in CLL and RT are shown. Center line indicates median; box limits indicate upper and lower quartiles; whiskers indicate 1.5 × interquartile range; points indicate individual samples. *P* values were derived using a one-tailed Wilcoxon rank-sum test. **d**, Heat map showing the DEGs between CLL and RT identified by bulk RNA-seq. Samples used in the differential expression analysis (DEA) are indicated. The overlap of DEGs with DNA methylation changes, H3K27ac and ATAC peaks is shown on the right. Selected genes are annotated. **e**, Intersection of upregulated genes in RT compared to CLL in scRNA-seq analyses. **f**, epiCMIT evolution from CLL to RT. *P* values were derived by paired Wilcoxon signed-rank test. **g**, Summary of the main gene sets modulated in RT based on bulk RNA-seq. NES, normalized enrichment score; ROS, reactive oxygen species. **h**, Gene set enrichment plot for OXPHOS and BCR signaling (bulk RNA-seq). **i**, OXPHOS and BCR signaling scores depicted at single-cell level for case 12 (all time points together). RT and CLL cells are highlighted (left). Ridge plots show the OXPHOS and BCR score across clusters (right). **j**, OXPHOS and BCR signaling scores of CLL and RT cells of patient 12 across time points by scRNA-seq. **k**, Distribution of OXPHOS and BCR signaling scores at a single-cell level across different time points of nine cases included in the study of Penter et al.^43^. Center line indicates median; box limits indicate upper and lower quartiles; whiskers indicate 1.5 × interquartile range; points indicate outliers. B, peripheral blood; M, bone marrow. *Sample collected under treatment with ibrutinib.

**Extended Data Fig. 9:**
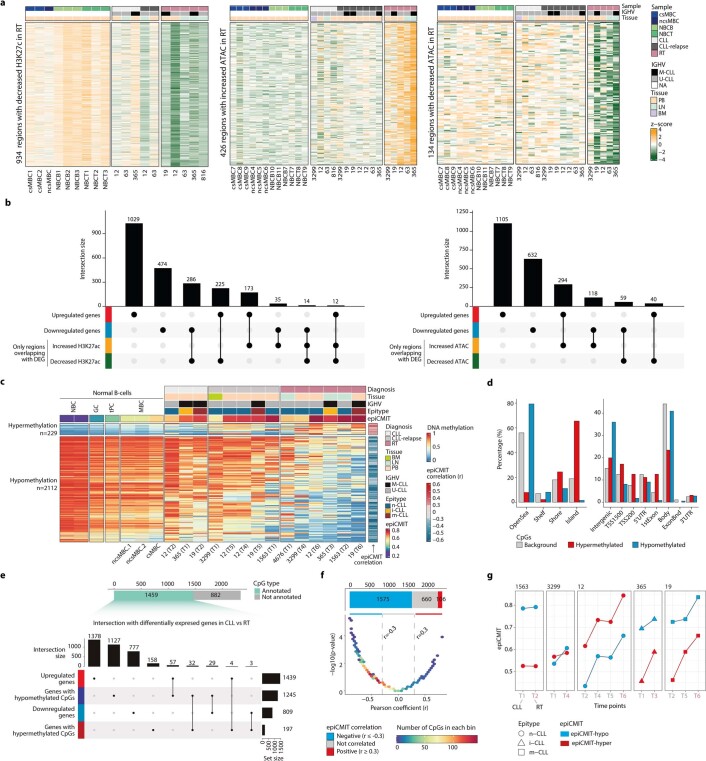
Epigenomic characterization of RT. **a**. Heatmaps showing the regions with decreased H3K27ac, increased ATAC, and decreased ATAC levels, respectively, in RT. **b**. Overlap of differentially expressed genes by bulk RNA-seq with regions with increased or decreased H3K27ac and ATAC levels, respectively. **c**. Heat map showing differentially methylated CpGs (DMC) between CLL and RT. Normal B cells, CLL, CLL at relapse, and RT samples are shown separately with different biological information on top. The correlation of each CpG with the epiCMIT is depicted on the right. To note, the epiCMIT is associated with the gain and loss of methylation upon cell division, but its transformation to 0-1 scale (for interpretability purposes) makes it anticorrelated with hypomethylation, as the epiCMIT=max{epiCMIT-hyper, epiCMIT-hypo}, being the epiCMIT-hyper=hypermethylation, and the epiCMIT-hypo=1-hypomethyaltion at relevant CpGs, as originally reported^49^. **d**. Genomic enrichment over the background for hyper- and hypomethylated CpGs in CLL vs RT. **e**. DMC distribution based on their genetic annotation and their intersection with differentially expressed genes by bulk RNA-seq analyses. **f**. DMC distribution based on the correlation of each CpG with the epiCMIT and their p values. CpGs were piled up in color-coded bins based on the number of CpGs in each bin to avoid overplotting. **g**. epiCMIT evolution in longitudinal CLL and RT samples, with the epiCMIT-hyper and epiCMIT-hypo scores depicted separately (RT samples being the last time point labeled in rose). The epiCMIT score used to compare among samples is the greater of the two (hyper and hypo).

To understand the transcriptomic evolution from CLL to RT and its epigenomic regulation, we integrated genome-wide profiles of DNA methylation, chromatin activation (H3K27ac) and chromatin accessibility (ATAC-seq) with bulk RNA-seq and scRNA-seq of multiple longitudinal samples of six patients treated with BCR inhibitors (Fig. 1a). The DNA methylome of RT mainly reflected the naive and memory-like B cell derivation of their CLL counterpart, whereas chromatin activation and accessibility were remarkably different upon transformation (Fig. 4a). We identified 150 regions with increased H3K27ac and 426 regions that gained accessibility in RT (Fig. 4b, Extended Data Fig. 9a and Supplementary Tables 7 and 8). These de novo active regions were enriched in transcription factor (TF) families different from those known to modulate the epigenome of CLL^44^. Among them, 24 were enriched and upregulated in RT (Supplementary Table 7). The top TF was TEAD4, which activates genes involved in oxidative phosphorylation (OXPHOS) through the mTOR pathway^45^ and co-operates with MYCN^46^. Additional TFs were related to MYC (MAZ), proliferation/cell cycle (E2F family) or IRF family, among others (Fig. 4c). Notably, high IRF4 levels seem to attenuate BCR signaling in CLL^47^, whereas they are necessary to induce MYC target genes, OXPHOS and glycolysis in activated healthy B cells^48^.

**Extended Data Fig. 10:**
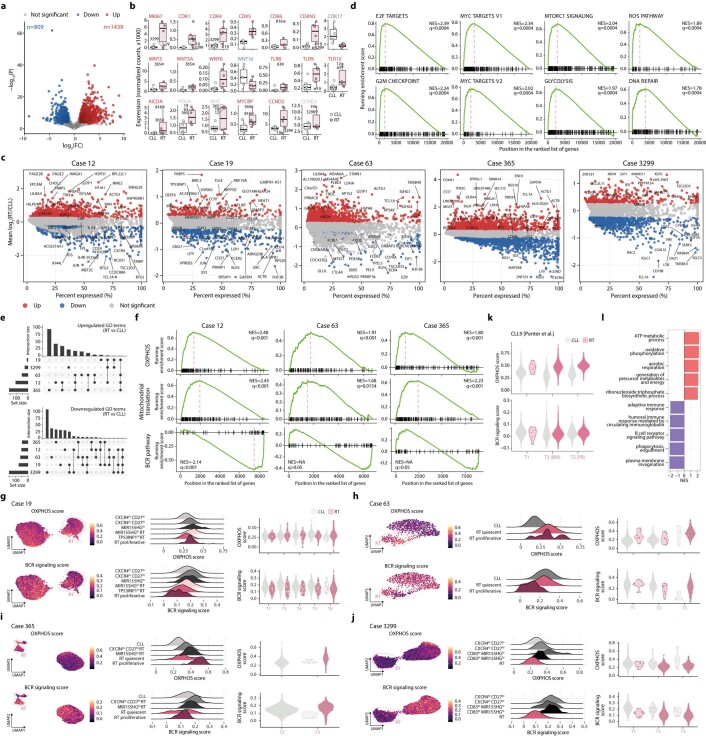
Transcriptomic characterization of RT. **a**. Volcano plot of the differential expression analysis (RT vs CLL, bulk RNA-seq). **b**. Expression levels of selected genes in CLL and RT according to bulk RNA-seq. center line, median; box limits, upper/lower quartiles; whiskers, 1.5×interquartile range; points, individual samples. **c**. Differentially expressed genes (RT vs CLL) for each case by scRNA-seq. **d**. GSEA plots of selected hallmark gene sets according to bulk RNA-seq analyses. NES, normalized enrichment score. **e**. UpSet plots highlighting the intersections of the case-specific upregulated (top) and downregulated (bottom) GO terms in RT by scRNA-seq. **f**. GSEA plots for the terms oxidative phosphorylation (OXPHOS), mitochondrial translation, and BCR signaling pathway for cases 12, 63, and 365 based on scRNA-seq. **g-j**. scRNA-seq-derived UMAP visualization of tumor cells from all time points colored by OXPHOS and BCR signaling score (left). Ridge plots showing the same scores across clusters (middle). Violin plots displaying the same scores across time points, stratified by CLL and RT clusters (right). **k**. Violin plots displaying the OXPHOS and BCR signaling scores across time points, stratified by CLL and RT clusters, in case CLL9 from Penter et al^43^. **l**. GSEA between RT and CLL cells of patient CLL9 from Penter et al.^43^.

The RNA-seq analysis, excluding cases 19 and 3,299 (RT-PLL) due to their intermediate transcriptomic profile, identified 2,248 differentially expressed genes (DEGs) between RT and CLL (1,439 upregulated and 809 downregulated) (Fig. 4a,d,e, Extended Data Fig. 10a and Supplementary Tables 11 and 23). A remarkable fraction of upregulated/downregulated genes overlapped with regions with the respective increase/decrease of H3K27ac (20%) and chromatin accessibility (16%) at RT (Fig. 4d and Extended Data Fig. 9b). Contrarily, only 4% of the DEGs overlapped with any of the 2,341 differentially methylated CpGs (DMCs) between RT and CLL, emphasizing the limited effect of DNA methylation on gene regulation^49^. Most DMCs were hypomethylated at RT (2,112 of 2,341; 90%), found in open sea and intergenic regions and correlated with the proliferative history of the cells measured by the epiCMIT score^49^ (1,681; 72%), which increased during CLL evolution and at RT (Fig. 4d,f, Extended Data Fig. 9c–g and Supplementary Table 6).

Genes upregulated in RT involved pathways that seem independent of BCR signaling such as Wnt (*WNT5A* and others)^50^, Toll-like receptors (*TLR9* among others)^51^ and a number of cyclin-dependent kinases. Downregulated genes included, among others, *CXCR4*, *HLA-A/B* and chromatin remodelers also targeted by genetic alterations in some cases (Fig. 4d and Extended Data Fig. 10b,c). Gene sets modulated by gene expression in RT were in harmony with the identified chromatin-based changes and included upregulation of E2F targets, G2M checkpoints, MYC targets, MTORC1 signaling, OXPHOS, mitochondrial translation, glycolysis, reactive oxygen species and DNA repair pathways, among others. In addition, RT showed downmodulation of BCR signaling (Fig. 4g,h, Extended Data Fig. 10d and Supplementary Table 11). The OXPHOS^high^–BCR^low^ pattern observed by bulk RNA-seq in RT was further refined using scRNA-seq: two of five tumors had OXPHOS^high^–BCR^low^ (12 and 63, although the latter showed some intercluster variability), the two M-CLL carrying IGLV3–21^R110^ had RT with BCR expression similar to CLL and were OXPHOS^high^–BCR^normal^ (365) or OXPHOS^normal^–BCR^normal^ (19) and the RT-PLL (3,299) was OXPHOS^low^–BCR^low^ (Fig. 4i, Extended Data Fig. 10e–j and Supplementary Table 23). In addition, the scRNA-seq analysis showed that the OXPHOS/BCR profiles of RT were already identified in the early dormant RT cells, suggesting that they might represent an intrinsic characteristic of RT cells rather than being modulated by BCR inhibitors (Fig. 4j and Extended Data Fig. 10g–j). To expand these observations, we measured the expression of OXPHOS and BCR pathways in the scRNA-seq dataset from Penter et al.^43^. Case CLL9, which developed RT in the absence of any therapy, showed a remarkably higher OXPHOS and slightly lower BCR expression at time of RT compared to CLL (Fig. 4k and Extended Data Fig. 10k,l).

Overall, the epigenome and transcriptome of RT converge to an OXPHOS^high^–BCR^low^ axis reminiscent of that observed in the de novo DLBCL subtype characterized by high OXPHOS (DLBCL-OXPHOS) and insensitive to BCR inhibition^52–54^. This axis might explain the selection and rapid expansion of small RT subclones under therapy with BCR inhibitors.

### OXPHOS and BCR activity in RT

**Fig. 5:**
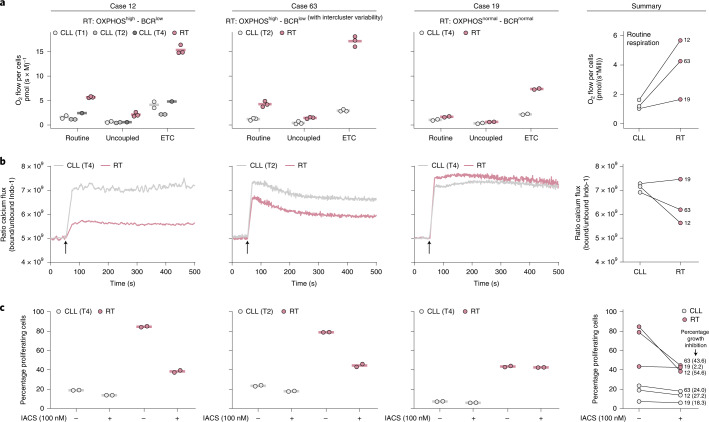
Cellular respiration, BCR signaling and OXPHOS inhibition in RT cells. **a**, Oxygen consumption of intact CLL and RT cells of three patients at routine respiration (routine), oligomycin-inhibited leak respiration (uncoupled) and uncoupler-stimulated ETC. Each dot represents a technical replicate. The mean of the replicates is shown using a horizontal line (left). Summary of the routine respiration of CLL and RT cells of the three patients collapsed (right). **b**, Calcium kinetics of tumoral cells (CD19^+^, CD5^+^) upon stimulation with 4-hydroxytamoxifen (4-OHT) and anti-BCR (black arrow). Basal calcium was adjusted at 5 × 10^9^ Indo-1 ratio for 60 s before cell stimulation with F(ab′)2 anti-human IgM + H_2_O_2_ at 37 °C. Then, Ca^2+^ flux was recorded up to 500 s (left). Summary of the calcium release after BCR stimulation of CLL and RT cells. Average mean fluorescence after stimulation is represented (right). **c**, Cell proliferation after 72-h incubation with or without IACS-010759 (IACS) at 100 nM. Percentage of proliferating cells was determined by carboxyfluorescein succinimidyl ester (CFSE) cell tracer. Two technical replicates of each sample were performed (left). Summary of the proliferation for each CLL and RT cells with or without IACS treatment after 72 h. The normalized percentage of growth inhibition is indicated (right).

We next validated experimentally the OXPHOS and BCR activity of RT in samples of patients 12, 19 and 63. Respirometry assays confirmed that OXPHOS^high^ RT cells (patients 12 and 63) had a 3.5-fold higher oxygen consumption at routine respiration and fivefold higher electron transfer system capacity (ETC) compared to CLL. In addition, OXPHOS^normal^ RT (patient 19) showed a routine oxygen consumption similar to CLL, although also had a relatively higher ETC than its CLL counterpart (Fig. 5a, Supplementary Fig. 3a–d and Supplementary Table 24). BCR signaling measured by Ca^2+^ mobilization upon BCR stimulation with IgM showed that BCR^low^ RT cells (patients 12 and 63) had a lower Ca^2+^ flux compared to CLL, which contrasted with the higher flux observed in the BCR^normal^ RT cells of patient 19, concordant with its IGLV3–21^R110^ mutation^27^ (Fig. 5b, Supplementary Fig. 4a,b and Supplementary Table 25).

To determine the biological effect of OXPHOS^high^ in RT, we performed in vitro proliferation assays using IACS-010759 (100 nM), an OXPHOS inhibitor that targets mitochondrial complex I (Supplementary Figs. 3e and 4c and Supplementary Table 25). OXPHOS^high^ RT (patients 12 and 63) had a higher proliferation at 72 h compared to OXPHOS^normal^ RT (patients 19) and all of them were higher than their respective CLL. OXPHOS inhibition resulted in a marked decrease in proliferation in OXPHOS^high^ RT (mean 49.1%), which contrasted with that observed in OXPHOS^normal^ RT (2.2% decrease) and CLL (23.2% decrease) (Fig. 5c and Supplementary Fig. 4d). Overall, these results confirm the role of OXPHOS^high^ phenotype in high proliferation of RT and suggest its potential therapeutic value in RT as proposed for other neoplasms^53–57^.

## Discussion

The genome of RT is characterized by a compendium of driver alterations in cell cycle, MYC, NOTCH and NF-κB pathways, frequently targeted in single catastrophic events and by the footprints of early-in-time, treatment-related, mutational processes, including the new SBS-RT potentially associated with bendamustine and chlorambucil exposure. A very early diversification of CLL leads to emergence of RT cells with fully assembled genomic, immunogenetic and transcriptomic profiles already at CLL diagnosis up to 19 years before the clonal explosion associated with the clinical transformation. RT cells have a notable shift in chromatin configuration and transcriptional program that converges into activation of the OXPHOS pathway and downregulation of BCR signaling, the latter potentially compensated by activating Toll-like, MYC and MAPK pathways^17,51,58,59^. The rapid expansion of RT subclones under treatment with BCR inhibitors is consistent with its low BCR signaling, except when carrying the IGLV3–21^R110^ and further supported by the increased number of subclones carrying unproductive immunoglobulin genes and the development of RT with plasmablastic differentiation, a cell type independent of BCR signaling^60^. Finally, we also uncovered that OXPHOS inhibition reduced the proliferation of RT cells in vitro, a finding worth exploring in future therapeutic strategies^55,57^.

In conclusion, our comprehensive characterization of CLL evolution toward RT has revealed new genomic drivers and epigenomic reconfiguration with very early emergence of subclones driving late stages of cancer evolution, which may set the basis for developing single-cell-based predictive strategies. Furthermore, this study also identifies new RT-specific therapeutic targets and suggests that early intervention to eradicate dormant RT subclones may prevent the future development of this lethal complication of CLL.

## Methods

### Consent and sample processing

Written informed consent was obtained from all patients. The study was approved by the Hospital Clinic of Barcelona Ethics Committee. Tumor DNA was extracted from tumor cells purified from fresh/cryopreserved mononuclear cells, frozen lymph nodes or formalin-fixed paraffin-embedded (FFPE) tissue (*n* = 1, CLL sample of patient 1,669). Germline DNA was obtained from the non-tumoral purified cell fraction in 12 cases. In two patients (1,523 and 4,675) who had received allogeneic stem-cell transplant before RT, germline DNA of the donor was also collected. All extractions were performed using appropriate QIAGEN kits (QIAamp DNA Blood Maxi kit, cat. no. 51194; QIAamp DNA Mini kit, cat. no. 51304; and AllPrep DNA/RNA FFPE kit, cat. no. 80234). Tumor RNA was obtained from tumor cells purified from fresh/cryopreserved mononuclear cells with TRIzol reagent (Invitrogen, cat. no. 15596026).

A specific flow cytometry analysis was conducted on peripheral blood samples of patient 12, which were stained with the Lymphocyte Screening Tube according to EuroFlow protocols (https://www.euroflow.org/protocols). At least 100,000 cells were acquired in a FACSCanto II instrument. Analysis was conducted using the Infinicyt 2.0 software. The sequential gating analysis was as follows: singlet identification in a FSC-W versus FSC-H plot; leukocyte identification in SSC-A versus CD45 (V500-C) plot and FSC-A versus SSC-A; lymphocytes identified as SSC-A low and CD45 high and back-gated in FSC-A versus SSC-A to exclude monocytes; in the lymphocyte gate, T cells were identified as CD3^+^ cells in SSC-A versus CD3 (APC) followed by sequentially distinguishing TCRγδ^+^ T cells, CD4 T cells and CD8 T cells; after excluding T cells, B cells were selected in a SSC-A versus CD19 (PE-Cy7), followed by inspection of CD19 (PECy7) versus CD20 (PacB), CD5 (PerCPCy5.5) versus CD20 (PacB) and CD20 (PacB) versus CD38 (APC-H7) plots to evaluate the expression of these B cell markers and the assignation of κ and λ expression in a plot of IgK (PE) versus IgL (FITC); after excluding B cells, natural killer cells were identified in a SSC-A versus CD56 (PE) plot followed by SSC-A versus CD38 (APC-H7) plot.

### WGS and WES

#### Library preparation and sequencing

All samples available were subjected to WGS except the FFPE CLL, which was analyzed by whole-exome sequencing (WES). WGS libraries were performed using the Kapa Library Preparation kit (Roche, cat. no. 07961901001), TruSeq DNA PCR-Free kit (Illumina, cat. no. 20015963) or TruSeq DNA Nano protocol (Illumina, cat. no. 20015965) and sequenced on a HiSeq 2000/4000/X Ten (2 × 126 bp or 2 × 151 bp) or NovaSeq 6000 (2 × 151 bp) instrument (Illumina). WES was performed using the SureSelect Human All Exon V5 (Agilent Technologies, cat. no. 5190-6209 and G9611B) coupled with a KAPA Hyper Prep kit (Roche, cat. no. 07962363001) for the DNA pre-capture library. Sequencing was performed on a HiSeq 2000 (2 × 101 bp). We also included WGS of three published CLL/germline pairs (patients 12, 19 and 63)^28^ (Supplementary Table 1).

#### General considerations

Overall, 12 patients had a complete dataset (germline, CLL and RT samples), 6 patients lacked germline DNA and 1 patient had only the RT sample (case 4,676). We conducted tumor versus normal analyses in cases with a complete dataset. For the six patients lacking the germline sample, we used the CLL samples as ‘normal’ to identify SNV acquired at RT for mutational signature analyses. In addition, tumor-only analyses were conducted in these CLL and RT samples, as well as in the patient with only a RT sample available, to identify driver gene mutations and genome-wide CNAs (Supplementary Table 1).

#### Read mapping and quality control

Reads were mapped to the human reference genome (GRCh37) using the BWA-MEM algorithm (v.0.7.15)^61^. BAM files were generated and optical/PCR duplicates flagged using biobambam2 (v.2.0.65, https://gitlab.com/german.tischler/biobambam2). FastQC (v.0.11.5, www.bioinformatics.babraham.ac.uk/projects/fastqc) and Picard (v.2.10.2, https://broadinstitute.github.io/picard) were used to extract quality control metrics. Mean coverage was 33× and 119× for WGS and WES, respectively (Supplementary Table 1).

#### Immunoglobulin gene characterization

Immunoglobulin gene rearrangements were characterized using IgCaller (v.1.2)^62^. The rearranged sequences obtained were reviewed on the Integrative Genomics Viewer (IGV; v.2.9.2)^63^ and annotated using IMGT/V-QUEST (https://www.imgt.org/IMGT_vquest) and ARResT/AssignSubsets (http://bat.infspire.org/arrest/assignsubsets).

#### Tumor versus normal SNVs and indel calling

SNVs were called using Sidrón^28^, CaVEMan (cgpCaVEManWrapper, v.1.12.0)^64^, Mutect2 (Genome Analysis Toolkit (GATK) v.4.0.2.0)^65^ and MuSE (v.1.0 rc)^66^ and normalized using bcftools (v.1.8)^67^. Variants detected by CaVEMan with more than half of the mutant reads clipped (CLPM > 0) and with supporting reads with a median alignment score (ASMD) <90, <120 or <140 for sequencing read lengths of 100, 125 or 150 bp, respectively, were excluded. Variants called by Mutect2 with MMQ < 60 were eliminated. Mutations detected by at least two algorithms were considered. Short insertions/deletions (indels) were called by SMuFin (v.0.9.4)^68^, Pindel (cgpPindel, v.2.2.3)^69^, SvABA (v.7.0.2)^70^, Mutect2 (GATK v.4.0.2.0)^65^ and Platypus (v.0.8.1)^71^. The somaticMutationDetector.py script (https://github.com/andyrimmer/Platypus/blob/master/extensions/Cancer/somaticMutationDetector.py) was used to identify somatic indels called by Platypus. Indels were left-aligned and normalized using bcftools^67^. Indels with MMQ < 60, MQ < 60 and MAPQ < 60 for Mutect2, Platypus and SvABA, respectively, were removed. Only indels identified by at least two algorithms were retained. Annotation of mutations was performed using snpEff/snpSift (v.4.3t)^72^ and GRCh37.p13.RefSeq as a reference. This approach showed a 93% specificity and 88% sensitivity when benchmarked against the mutations found at a VAF >10% in our previous high-coverage NGS study^73^.

#### Tumor-only SNVs and indel calling

Tumor-only variant calling was restricted to coding regions of 243 genes described as drivers in CLL and other B cell lymphomas (Supplementary Table 10). Mini-BAM files were obtained using Picard tools and variant calling was performed using Mutect2 (GATK v.4.0.4.0)^65^, VarScan2 (v.2.4.3)^74^, VarDictJava (v.1.4)^75^, LoFreq (v.2.1.3.1)^76^, outLyzer (v.1.0)^77^ and freebayes (v.1.1.0, https://github.com/freebayes/freebayes). Variants were normalized using bcftools (v.1.9)^67^ and annotated using snpEff/snpSift (v.4.3t)^72^. Only non-synonymous variants that were identified as PASS by ≥2 algorithms were considered. Variants reported in 1000 Genomes Project, ExAC or gnomAD with a population frequency >1% or reported as germline in our ICGC database of 506 WES/WGS^28^ were considered as polymorphisms.

#### Tumor versus normal CNA calling

CNAs were called using Battenberg (cgpBattenberg, v.3.2.2)^78^ and ASCAT (ascatNgs, v.4.1.0)^79^. CNAs within any of the immunoglobulin loci were not considered. We used the tumor purities obtained by Battenberg in downstream analyses. The median tumor cell content was 91.5% (Supplementary Table 1).

#### Tumor-only CNA calling

CNAs were extracted using CNVkit (v.0.9.3)^80^. CNAs <500 kb, with an absolute log_2_ copy ratio (log_2_CR) < 0.3 or located within any of the immunoglobulin loci were removed. CNAs were classified as gains if log_2_CR > 0.3, deletions if log_2_CR < −0.3, high-copy gains if log_2_CR > 1.1 and homozygous deletions if log_2_CR < −1.1. The log_2_CR cutoff was set to 0.15 for two samples with low tumor cell content (102-01-01TD and 4690-03-01BD). To avoid a high segmentation of the CNA profile, CNAs belonging to the same class were merged if they were separated by <1 Mb and had an absolute log_2_CR difference <0.25.

#### Array-based CNA calling in FFPE

CNAs were examined in the FFPE CLL sample using the Oncoscan CNV FFPE Assay kit (Thermo Fisher Scientific, cat. no. 902695) and analyzed using Nexus 9.0 software (Biodiscovery).

#### Tumor versus normal SV calling

SVs were extracted using SMuFin (v.0.9.4)^68^, BRASS (v.6.0.5)^81^, SvABA (v.7.0.2)^70^ and DELLY2 (v.0.8.1)^82^. SVs identified were intersected considering a window of 300 bp around break points. We kept for downstream analyses the SVs identified by at least two programs if at least one of the algorithms called the alteration with high quality (MAPQ ≥ 90 for BRASS, MAPQ = 60 for SvABA and DELLY2). In addition, IgCaller (v.1.2)^62^ was used to call SVs within any of the immunoglobulin loci. All SVs were visually inspected using IGV^63^. SVs were categorized into simple or complex events. Chromothripsis^83^ was defined as ≥7 oscillating changes between two or three copy number states or the presence of >7 SV break points occurring in a single chromosome and supported by additional criteria^83,84^. Chromoplexy was determined by the presence of ≥3 chained chromosomal rearrangements, where chains were identified using a window of 50 kb^85,86^. Cycles of templated insertions were defined as copy number gains in ≥3 chromosomes linked by SVs^87^. Breakage-fusion bridge cycles were defined as patterns of focal copy number increases and fold-back inversions, together with telomeric deletions. Chains of rearrangements having >2 SVs and not fulfilling any of the previous criteria were classified as ‘other complex events’. Chromothripsis and ‘other complex events’ were subcategorized according to the number of chromosomes involved. The longitudinal nature of our dataset allowed us to refine the obtained classification based on the presence of the involved alterations in each time point analyzed.

#### Patients who underwent allogenic stem-cell transplant

In these patients, we conducted tumor versus patient’s germline and tumor versus donor’s germline variant calling in parallel. Only the intersection of variants identified was considered.

#### Rescue of alterations based on longitudinal information

SNVs called in one sample were automatically added to the samples of additional time point(s) if at least one high-quality read with the mutation was found in the BAM file (alleleCounter v.4.0.0, parameters: min_map_qual = 35; and min_base_qual = 20). Similarly, indels and SVs detected in one sample were added in the additional time point(s) if any of the algorithms detected the alteration, regardless of its filters.

#### WGS-based subclonal reconstruction

A Markov chain Monte Carlo sampler for a Dirichlet process mixture model was used to infer putative subclones, to assign mutations to subclones and to estimate the subclone frequencies in each sample from the SNV read counts, copy number states and tumor purities (Supplementary Table 17)^78,88^. Clusters with <100 mutations were excluded. The phylogenetic relationships between subclones were identified following the ‘pigeonhole principle’, which was relaxed using a case-specific ‘tolerated error’^88^. Clusters not assigned to the reconstructed phylogenetic tree were excluded. Fish plots were generated using the TimeScape R package (v.1.6.0). The CCF of indels was calculated integrating read counts, CNAs and tumor purity^89^. Driver indels subjected to validation by scDNA-seq and/or relevant to the tumor phylogeny were manually assigned to subclones. Similarly, driver CNAs relevant to the phylogeny were manually assigned. Seven SNVs found in *TP53*/*ATM* overlapping with CNAs were manually assigned to the most likely subclone as they were not automatically assigned by the Dirichlet process and were subjected to scDNA-seq (Supplementary Table 9).

### Mutational signatures

We studied mutational signatures acting genome-wide and in localized regions (inter-mutation distance ≤1Kb)^29,32^. We integrated the mutations identified in this CLL/RT cohort together with those of 147 CLL treatment-naive samples (ICGC-CLL)^28^ and 27 new CLL collected at relapse post-treatment (mean coverage 31.5×; Supplementary Table 15). The WGS of these two additional cohorts was (re-)analyzed using our current bioinformatic pipeline (Supplementary Table 12). Mutational signatures were analyzed for SNVs or single-base substitutions (SBSs) according to their 5′ and 3′ flanking bases following three steps^30^:

1. Extraction: de novo signature extraction was performed using a hierarchical Dirichlet process (HDP, v.0.1.5; https://github.com/nicolaroberts/hdp), SignatureAnalyzer (v.0.0.7)^90^, SigProfiler (SigProfilerExtractor, v.1.0.8)^32^ and sigfit (v.2.0.0; https://github.com/kgori/sigfit). HDP was run with four independent posterior sampling chains, followed by 20,000 burn-in iterations and the collection of 200 posterior samples off each chain with 200 iterations between each. SigProfiler was run with 1,000 iterations and a maximum of ten extracted signatures. Similarly, sigfit was run to extract five signatures with 10,000 burn-in iterations and 20,000 sampling iterations.
2. Assignment: each extracted signature was assigned to a given COSMIC signature (v.3.2)^32^ if their cosine similarity was >0.85. Otherwise, the extracted signature was decomposed into ‘*n*’ COSMIC signatures using an expectation maximization (EM) algorithm^91^. The EM algorithm was first run using the COSMIC signatures identified in the previous step. If their cosine similarity was <0.85, we ran the EM algorithm, including all signatures reported in COSMIC and by Kucab et al.^33^ (55 mutational signatures related to environmental agents). Three exceptions were made: (1) we combined two HDP signatures that together constituted COSMIC signature SBS5 to avoid splitting of signatures (Extended Data Fig. 4a); (2) APOBEC signatures (SBS2 and SBS13) were favored to be assigned to one of the signatures extracted by HDP and SignatureAnalyzer although it was not the best EM solution probably because they were only found in one sample, which impaired a clean extraction of the signatures (Extended Data Fig. 4f); and (3) one signature extracted by HDP and SignatureAnalyzer was directly assigned to the mutational signature associated with ganciclovir treatment^35^ (cosine similarity 0.987 and 0.993, respectively) (Extended Data Fig. 4). The new SBS-RT extracted by HDP was considered for downstream analyses as it had less background noise than the one extracted by SignatureAnalyzer, favoring a higher specificity during the fitting step. Similarly, the SBS-ganciclovir extracted by HDP was used in downstream analyses (Extended Data Fig. 4). We also performed a detailed review to remove signatures susceptible of being originated due to sequencing artifacts (Supplementary Table 13).
3. Fitting: we used a fitting approach (MutationalPatterns, v.3.0.1) to measure the contribution of each mutational signature in each sample. Based on (1) the de novo identification of the therapy-related SBS-ganciclovir and (2) that two patients received melphalan before RT, the mutational signature associated with melphalan therapy^34^ was also included in this step. To avoid the so-called inter-sample bleeding effect^30^, we iteratively removed the less-contributing signature if its removal decreased the cosine similarity between the original and reconstructed 96-profile <0.01 (ref. ^32^). SBS1 and SBS5 were added if addition improved the cosine similarity^32^. Similarly, SBS9 was added in CLL/RT samples classified as M-CLL if addition improved the cosine similarity. We also ran mSigAct (v.2.1.1; https://github.com/steverozen/mSigAct) to confirm the presence/absence of SBS-melphalan (Supplementary Table 15). To assess the contribution of each signature to each subclone we followed the same fitting strategy but (1) considered only the signatures that were present in the corresponding sample and (2) removed the final step of adding SBS9 in M-CLL to avoid its addition in multiple subclones with low evidence.

#### Genomic locations and strand bias

We assessed the contribution of SBS-RT to coding SNVs in RT subclones (also including cases in which the CLL sample was used as a ‘germline’) by calculating the probability that a given mutation was caused by SBS-RT. To perform this calculation, we considered the signatures present in the subclone/sample and their signature profile^92^. The reference epigenomes of CLL^44^ were used to explore the contribution of the mutational processes in different regulatory regions. We simplified the described chromatin states in four categories: heterochromatin (H3K9me3_Repressed, Heterochromatin Low_Signal), polycomb (Posied_Promoter, H3K27me3_Repressed), enhancer/promoter (Active_Promoter, Strong_Enhancer1, Weak_Promoter, Weak_Enhancer, Strong_Enhancer) and transcription (Transcription_Transition, Weak_Transcription, Transcription_Elongation). We also mapped the activity of mutational processes in early/late replication regions of the genome considering peaks/valleys of early/late replication as those regions of ≥1 kb with absolute replication timing >0.5 (ref. ^93^). All SNVs of the CLL and RT subclones were classified in any of the four chromatin states and early/late replication regions before fitting mutational signatures. A cutoff of 0.005 was used to remove the less-contributing signature during the fitting step. We also generated replication and transcriptional strand bias profiles of the RT-specific mutations using the MutationalPatterns R package^34^. The replication strand was annotated based on the left/right replication direction of the timing transition regions^94^. The transcriptional strand was annotated using the TxDb.Hsapiens.UCSC.hg19.knownGene R package (v.3.2.2). Finally, kataegis was defined as a genomic region having six or more mutations with an average inter-mutation distance ≤1 kb.

### High-coverage, UMI-based gene mutation analysis

#### Data generation

A high-coverage, UMI-based NGS was performed to track 77 mutations identified by WGS (Supplementary Table 18). Molecular-barcoded and target-enriched libraries were prepared using a Custom CleanPlex UMI NGS Panel (Paragon Genomics) and CleanPlex Unique Dual-Indexed PCR Primers for Illumina (Paragon Genomics, cat. no. 716011 and 716013). Libraries were sequenced on a MiSeq and/or NextSeq 2000 instrument (2 × 150 bp, Illumina).

#### Data analysis

Raw reads were trimmed using cutadapt (https://cutadapt.readthedocs.io; v.1.15 with parameters: -g CCTACACGACGCTCTTCCGATCT -a AGATCGGAAGAGCACACGTCTGAA -A AGATCGGAAGAGCGTCGTGTAGG -G TTCAGACGTGTGCTCTTCCGATCT -e 0.1 -O 9 -m 20 -n 2). Trimmed FASTQ reads were converted to unmapped BAM using Picard’s FastqToSam tool (v.2.10.2). UMI information was extracted and stored as a tag using fgbio ExtractUmisFromBam (http://fulcrumgenomics.github.io/fgbio/; v.1.3.0 with parameters: –read structure = 16M+T 16M+T, –single-tag = RX, –molecular-index-tags = ZA ZB). Template read was converted to FASTQ with Picard’s SamToFastq. Template reads were mapped against the human reference genome (GRCh37) and reads were merged with the UMI information using Picard’s MergeBamAlignment. Finally, reads were grouped by UMI and a consensus was called using fgbio GroupReadsByUmi (parameters were –strategy = adjacency, –edits = 1, –min-map = 10) and CallMolecularConsensusReads (parameters were –min-reads = 3), respectively. A minimum of three reads was required to create a UMI-based final read. Final reads were converted back to FASTQ using Picard’s SamToFastq and mapped against the reference genome using BWA-MEM (v.0.7.15)^61^. Mean coverage was determined using Picard’s CollectTargetedPcrMetrics (parameters: CLIP_OVERLAPPING_READS = true, MINIMUM_MAPPING_QUALITY = 15 MINIMUM_BASE_QUALITY = 15). Read counts were collected at all targeted genomic positions for all samples using bcftools mpileup (v.1.8, parameters: -B -Q 13 -q 10 -d 100,000 -a FORMAT/DP,FORMAT/AD,FORMAT/ADF,FORMAT/ADR -O v)^67^. Allele positions lacking mutations by WGS were used to model the background sequencing noise, which was unified according to the trinucleotide context of each possible mutation. Mutations of interest were annotated as high confidence when their frequency was above the background noise with a probability of 95%.

### High-coverage immunoglobulin gene characterization

#### DNA-based

The LymphoTrack IGHV Leader Somatic Hypermutation Assay Panel, MiSeq (Invivoscribe Technologies, cat. no. 71210069) was performed in samples of two patients (Supplementary Table 21). Libraries were sequenced on a MiSeq instrument (2 × 301 bp, Illumina). Clonotypes were defined as IGHV-IGHD-IGHJ gene rearrangements with the same IGHV gene and IGH CDR3 amino acid sequence within a sample. Clonotypes with different nucleotide substitutions within the FR1-CDR1-FR2-CDR2-FR3 sequence of the rearranged IGHV gene were defined as subclones. Raw FASTQ files were trimmed using Trimmomatic (v.0.36)^95^ to keep only high-quality reads and bases (parameters were LEADING:30 TRAILING:30 SLIDINGWINDOW:4:30 MINLEN:100). Trimmed, paired-end FASTQ files were analyzed using the LymphoTrack Software, MiSeq (v.2.3.1, Invivoscribe Technologies, cat. no. 75000009), which combines forward and reverse reads to generate full-length sequences. Identical full-length sequences were grouped and reported together with their cumulative frequency. The reported full-length sequences were annotated using IMGT/HighV-QUEST (v.1.8.3; https://www.imgt.org/HighV-QUEST). Finally, we (1) selected the sequences that belonged to the dominant productive clonotype; (2) kept only sequences with complete V-region (missing bases and indels within the V-region were not allowed); and (3) merged sequences that shared the exact V-region nucleotide sequence.

#### RNA-based

For patient 12, cryopreserved samples collected at four different time points were thawed and malignant cells were enriched using the The EasySep Human B Cell Enrichment kit II without CD43 depletion (Stemcell Technologies, cat. no. 17923). Next, 1–2 million tumor cells were used to perform the Omniscope BCR VDJ sequencing assay (https://www.omniscope.ai). Cells were lysed and the RNA was reverse transcribed to complementary DNA with UMIs before amplification of the V(D)J region using BCR-specific multiplex PCR. Following sequencing, reads were aligned using STARsolo (v.2.7.9a; https://github.com/alexdobin/STAR/blob/master/docs/STARsolo.md) to the hg38 human genome. IGV^63^ was used to review and quantify the mutation of interest (chr14:106714886C>T).

### DNA methylation

#### Data generation and processing

DNA methylation data of 39 samples was generated using EPIC BeadChips (Illumina). These samples included different healthy B cell subpopulations (naive B cells (NBCs), *n* = 2; germinal center B cells (GCs), *n* = 1; memory B cells (MBCs), *n* = 3; tonsillar plasma cells (tPCs), *n* = 1); CLL samples without evidence of RT (*n* = 12) and longitudinal CLL/RT samples (*n* = 20) (Supplementary Table 6). R and core Bioconductor packages, including minfi (v.1.34.0)^96^, were used to integrate and normalize DNA methylation data^49^. We removed non-CpG probes, CpGs representing single nucleotide polymorphisms, CpGs with individual-specific methylation previously reported in B cells, CpGs in sex chromosomes and CpGs with a detection *P* value >0.01 in >10% of the samples. The data were normalized using the SWAN algorithm and CpGs were annotated using the IlluminaHumanMethylationEPICanno.ilm10b4.hg19 package (v.0.6). Tumor cell content of each sample was inferred from DNA methylation^49^ and samples with a tumor cell content <60% were excluded. After all filtering criteria, we retained 33 samples (NBCs, *n* = 2; GCs, *n* = 1; MBCs, *n* = 3; tPCs, *n* = 1; CLL controls, *n* = 12; CLL/RT samples, *n* = 14 (six patients); Supplementary Table 6).

#### Differential analyses, CLL epitypes and epiCMIT

We compared the DNA methylation status of each CpG to the mean of such CpGs in NBCs to calculate the number of hyper- and hypomethylation changes per CLL/RT sample. Changes in each sample were defined based on a minimum difference of 0.25 methylation. To perform a differential analysis between CLL and RT, we compared the DNA methylation of each CpG in each CLL sample (first available time point used) versus their respective RT sample. Differentially methylated CpGs were considered as those showing a minimum difference of 0.25 in at least four of the five longitudinal cases of RT versus CLL analyzed (Supplementary Table 6). The epigenetic subtypes (epitypes) and epiCMIT score for each CLL and RT sample were calculated^49^.

### ChIP-seq of H3K27ac and ATAC-seq

#### Data generation

ChIP-seq of H3K27ac and ATAC-seq data were generated as described in http://www.blueprint-epigenome.eu/index.cfm?p=7BF8A4B6-F4FE-861A-2AD57A08D63D0B58 (antibody anti H3K27ac, Diagenode, cat. no. C15410196/pAb-196-050, lot A1723-0041D; Supplementary Tables 7 and 8). Libraries were sequenced on Illumina machines aiming at 60 million reads/sample (Supplementary Tables 7 and 8).

#### Read mapping and initial data processing

FASTQ files were aligned to the reference genome (GRCh38) using BWA-ALN (v.0.7.7, parameter: -q 5)^61^, duplicated reads were marked using Picard tools (v.2.8.1) and low-quality and duplicated reads were removed using SAMtools (v.1.3.1, parameters: -b -F 4 -q 5 -b -F 1,024)^67^. PhantomPeakQualTools (v.1.1.0) were used to generate wiggle plots and for extracting the predominant insert-size. Peaks were called using MACS2 (v.2.1.1.20160309, parameters for H3K27ac: -g hs -q 0.05 -keep-dup all -nomodel -extsize insert-size; parameters for ATAC-seq: -g hs -q 0.05–keep-dup all -f BAM –nomodel –shift −96 –extsize 200; no input control)^97^. Peaks with *q* values <1 × 10^−3^ were included for downstream analyses. For each mark separately, a set of consensus peaks, including regions within chromosomes 1–22 and present in published healthy B cells^44^ and CLL samples was generated by merging the locations of the separate peaks per individual sample. For ChIP-seq, the numbers of reads per sample per consensus peak were calculated using the genomecov function (bedtools, v.2.25.0). For ATAC-seq, the number of Tn5 transposase insertions per sample per consensus peak was calculated by first determining the estimated insertion sites (shifting the start of the first mate 4 bp downstream) before using the genomecov function. Variance stabilizing transformation (VST) values were calculated for all consensus peaks using DESeq2 (v.1.28.1)^98^, which were then corrected for the consensus SPOT score (the percentage of reads that fall within the consensus peaks) using the ComBat function (sva R package, v.3.36.0). To that purpose, the cell condition (tumor and different healthy B cell subtypes) was assigned to each sample and samples were clustered in 20 bins of 5% according to their consensus SPOT score. The bins on the extremes, which contained fewer than five samples, were joined with their neighboring bins to ensure that each bin contained five samples or more. PCA was generated using the corrected VST values of peaks that were present in more than one sample.

#### Detection of differential epigenetic regions and RT-specific changes

We first determined the regions with stable epigenetic profiles in the healthy B cell counterparts (NBCs and MBCs) by applying a threshold of s.d. < 0.8 with respect to the mean value. For all these NBC/MBC stable regions, we then calculated the log_2_FC between the mean of VST-corrected healthy B cell values and each of the tumor samples. Due to the data distribution variability, we applied slightly different thresholds of log_2_FC for each case (Supplementary Tables 7 and 8). To identify regions changing in RT for each case individually, we selected the regions that presented substantial epigenetic changes as compared to the normal counterpart and to the previous CLL (absolute log_2_FC > 1). The ATAC-seq RT-specific signature encompassed differential regions common in two or more cases of RT, whereas the H3K27ac RT-specific signature included differential regions common in three or more cases. Potential protein-coding target genes were assigned to each of the RT-specific regions using two strategies. To identify close target genes, we took the overlap with the regions of genes of interest adding 2 kb upstream of their transcription start site. To identify distant target genes, we used Hi-C data from the GM12878 cell line and selected all genes located within the same topologically associated domain as the region of interest. We only considered DEGs identified by bulk RNA-seq (Supplementary Tables 7 and 8).

#### Transcription factor analysis

Enrichment for TF-binding sites was analyzed in chromatin accessible regions within the RT-specific active chromatin regions. Accessible peaks were determined as regions with presence of ATAC peaks in two or more RT cases. Enrichment analysis of known TF-binding motifs was performed using the AME tool (MEME suite) considering the non-redundant *Homo sapiens* 2020 Jaspar database and applying one-tailed Wilcoxon rank-sum tests with the maximum score of the sequence, a 0.01 FDR cutoff and a background formed by reference GRCh38 sequences extracted from the consensus ATAC-seq peaks (91,671 regions). We then established the occupancy of these motifs in RT and CLL by calculating the percentage of the target RT-specific active regions and of the regions with increased H3K27ac in CLL, respectively, which contained these motifs. Finally, we selected TFs presenting an occupancy difference between RT and CLL ≥ 10% and overexpressed in RT (bulk RNA-seq, log_2_FC > 0, adjusted *P* value <0.01).

### Bulk RNA-seq

#### Data generation

Bulk RNA-seq data of six patients with paired CLL and RT samples were analyzed. Libraries were prepared using the TruSeq Stranded mRNA Library Prep kit (Illumina, cat. no. 20020595) or the Stranded mRNA Library Prep, Ligation kit (Illumina, cat. no. 20040534) and sequenced on a HiSeq 4000 (2 × 76 bp, Illumina) or NextSeq 2000 (2 × 100 bp, Illumina). All samples had a tumor purity ≥92% as assessed by flow cytometry (Supplementary Table 11).

#### Data analysis

Ribosomal RNA reads were filter out using SortMeRNA (v.4.3.2)^99^. Non-ribosomal reads were trimmed using Trimmomatic (v.0.38)^95^. Gene-level counts (GRCh38.p13, Ensembl release 100) were calculated using kallisto (v.0.46.1)^100^ and tximport (v.1.14.2). A paired DEA was conducted using DESeq2 (v.1.26.0)^98^. Adjusted *P* value <0.01 and absolute log_2_(fold change) > 1 were used to identify DEGs. Gene set enrichment analysis (GSEA) was conducted using a pre-ranked gene list ordered by −log_10_(*P*) × (sign of fold change) using the ‘GSEA’ function (clusterProfiler R package, v.3.14.3). We focused on C2 (curated) and Hallmark gene sets from the Molecular Signatures Database (v.7.4) with a minimal size of 10 and maximal size of 250. Gene ontology (GO) GSEA was conducted using the pre-ranked gene list as input of the ‘gseGO’ function (clusterProfiler) focusing on biological processes. Redundancy in the output list of GO terms was removed using the ‘simplify’ function (cutoff of 0.35).

### Single-cell DNA-seq

#### Data generation

scDNA-seq was performed for 16 samples of 4 patients using the Tapestri Platform (Mission Bio, cat. no. 191335) and a commercial 32-gene panel (Tapestri single-cell DNA CLL panel, Mission Bio, cat. no. MB53-0011_J01). Cryopreserved cells were thawed on 5 ml of fetal bovine serum (FBS; Fisher Scientific, cat. no. 10082147) and incubated at 37 °C for 5 min. Then, cells were washed twice with 1 ml phosphate buffered saline (PBS; Thermo Fisher, cat. no. 20012-019) with 4% bovine serum albumin (BSA; Miltenyi Biotec, cat. no. 130-091-376) and centrifuged at 400*g* for 4 min. Cell concentration and viability were verified by counting with a TC20T Automated Cell Counter (Bio-Rad Laboratories, cat. no. 1450102). After a final centrifugation step, supernatant was removed and cells were resuspended in an appropriate volume of Mission Bio cell buffer to obtain a final cell density of 3,000–4,000 cells μl^−1^. Encapsulation, lysis and barcoding of cells were performed following the exact manufacturer’s instructions. Afterwards, PCR products were digested and cleaned up with AMPure XP Reagent (Beckman Coulter, cat. no. 100-265-900), followed by quantification of PCR products using a High-Sensitivity dsDNA 1× Qubit kit (Qubit, Invitrogen, cat. no. Q32851). Final library preparation consisted of a Target Library PCR with the V2 Index Primer for ten cycles and a library cleanup with AMPure XP Reagent (Beckman Coulter). Quality control and final quantification were performed on an Agilent Bioanalyzer High Sensitivity chip (Agilent Technologies, cat. no. 5067-4626). Libraries were sequenced on a NovaSeq 6000 instrument (Illumina) aiming for 1,300 reads per cell (Supplementary Table 20).

#### Data analysis

FASTQ files were analyzed through the Tapestri Pipeline (v.1, Mission Bio), which trims adaptor sequences, aligns reads to the human genome (hg19) using BWA aligner, performs barcode correction, assigns sequence reads to cell barcodes and performs genotype calling using GATK (v.3.7). Loom files generated were analyzed using the Tapestri Insights (v.2.2, Mission Bio). For each patient (considering all time points together), genotypes with quality <30, read depth <10 or allele frequency <20% were marked as missing. Similarly, for each patient, variants genotyped in <50% of the cells or mutated in <1% of the cells were removed. Cells with <50% of genotypes present were removed. Mutations identified in bulk WGS analysis were used as a whitelist. A list of variants not identified in COSMIC and present at low frequency (1–10% of cells) in all samples analyzed by scDNA-seq was used to remove potential artifacts. The analysis was restricted to coding and splice-site mutations. Genotypes of the selected mutations were exported from Tapestri Insights and used as input of ∞SCITE (https://github.com/cbg-ethz/infSCITE)^101^. Genotypes were encoded as zero for wild-type, one for heterozygous mutation, two for homozygous mutation and three for missing data. ∞SCITE was used to find the mutation tree that best fitted the genotypes observed and to assign cells into subclones. ∞SCITE was run using a global sequencing error rate (false-positive rate) of 1%^102^, an estimated rate of non-mutated sites called as homozygous mutations of 0% and a patient-specific estimated rate of the allele dropout rate (false-negative rate). For each patient, the estimated rate of missed heterozygous mutations (dropout of the mutated allele) and the estimated rate of heterozygous mutations called as homozygous mutations (dropout of the normal allele) were calculated from germline single-nucleotide polymorphisms reported in gnomAD with a population frequency >1% and called as mutated in at least 75% of cells with a VAF per read count between 47% and 53% according to Tapestri Insights. Patient-specific allele dropout rates were calculated for all patients except for patient 365, which did not have any heterozygous polymorphisms fulfilling the previous criteria. In this case, we used an allele dropout rate of 0.07, which is within the range measured in the other cases. We ran ∞SCITE with and without considering *NOTCH1* mutations and manually curated the result of patient 3,299 carrying an *RPS15* mutation due to the high allele dropout rate observed in these genes (Supplementary Fig. 2). We ran ∞SCITE for each patient combining all time points and obtained time-point-specific subclone sizes by counting the cells assigned to each subclone in each sample^102^. Only cells uniquely assigned to one subclone were considered. Cells genotyped as wild-type for all selected mutations were considered as non-tumoral cells and were removed.

### Single-cell RNA-seq

#### Data generation

scRNA-seq was performed on longitudinal samples of five patients using three different approaches:

1. Smart-seq2: full-length scRNA-seq libraries were prepared for samples of patient 63 using the Smart-seq2 protocol^103^ with minor modifications. Single cells were sorted into 96-well plates containing the lysis buffer (0.2% Triton-100, 1 U μl^−1^ RNase inhibitor; Applied Biosystems, cat. no. N8080119). Reverse transcription was performed using SuperScript II (Thermo Fisher Scientific, cat. no. 18064014) in the presence of 1 μM oligo-dT30VN (IDT, cat. no. 22859789), 1 μM template-switching oligonucleotides (QIAGEN, cat. no. PER-YCO0075516) and 1 M betaine (Merck, cat. no. W422312-5KG-K). cDNA was amplified using the KAPA Hifi Hotstart ReadyMix (Kapa Biosystems, cat. no. 7958935001) and IS PCR primer (IDT, cat. no. 228597989), with 25 cycles of amplification. Following purification with Agencourt Ampure XP beads (Beckmann Coulter), product size distribution and quantity were assessed on a Bioanalyzer using a High Sensitivity DNA kit (Agilent Technologies). A total of 140 pg of the amplified cDNA was fragmented using Nextera XT (Illumina, cat. no. FC-131-1096) and amplified with Nextera XT indexes (Illumina, cat. no. 20027215). Products of each well of the 96-well plate were pooled and purified twice with Agencourt Ampure XP beads (Beckmann Coulter). Pooled sequencing was performed on a HiSeq 4000 (2x75bp, Illumina) to an average depth of 0.5 million reads per cell.
2. Cell hashing experiment and 10x Genomics: For each patient (12, 19, 365 and 3,299, experiment BCLLATLAS_10), samples obtained at different time points of the disease were labeled following a cell hashing protocol^104^. For each sample, 1–2 million cells were resuspended in 100 μl of cell staining buffer (BioLegend, cat. no. 420201) and incubated for 10 min at 4 °C with 5 μl of Human TruStain FcX Fc Blocking reagent (BioLegend, cat. no. 422302). Next, a specific TotalSeq-A antibody-oligo conjugate (BioLegend, TotalSeq-A anti-human Hashtag 1–8, cat. no. 394601, 394603, 394605, 394607, 394609, 394611, 394613 and 394615) was added and incubated on ice for 30 min. Cells were then washed three times with cold PBS-0.05% BSA and centrifuged for 5 min at 500*g* at 4 °C. Finally, cells were resuspended in an appropriate volume of 1× PBS-0.05% BSA to obtain a final cell concentration of 500–1,000 cells μl^−1^, suitable for 10x Genomics scRNA-seq. An equal volume of hashed cell suspension from each of the conditions was mixed and filtered with a 40-µm strainer (pluriSelect, cat. no. 43-10040-70). Cell concentration was verified by counting with a TC20 Automated Cell Counter (Bio-Rad Laboratories, cat. no. 1450102). Cells were partitioned into Gel Bead In Emulsions with a Target Cell Recovery of 10,000 total cells. Sequencing libraries were prepared using the Chromium Next GEM Single Cell 3′ GEM, Library & Gel Bead kit v.3.1 (10x Genomics, cat. no. 1000121) with some adaptations for cell hashing, as indicated in TotalSeq-A Antibodies and Cell Hashing with 10x Single Cell 3′ Reagent kit v.3.1 Protocol by BioLegend. Briefly, 1 µl of 0.2 µM HTO primer (IDT, Hashtag Oligonucleotides; GTGACTGGAGTTCAGACGTGTGC*T*C; *phosphorothioate bond) was added to the cDNA amplification reaction to amplify the hashtag oligonucleotides together with the full-length cDNAs. An SPRI selection cleanup was performed to separate messenger RNA-derived cDNA (>300 bp) from antibody-oligonucleotide-derived cDNA (<180 bp), as described in the above-mentioned protocol. 10x cDNA sequencing libraries were prepared following 10x Genomics Single Cell 3′ v.3.1 mRNA kit protocol, whereas HTO cDNAs were indexed by PCR as follows: 5 µl of purified hashtag oligonucleotide cDNA were mixed with 2.5 µl of 10 µM Illumina TruSeq D70X_s primer (IDT) carrying a different i7 index for each sample, 2.5 µl of SI primer (10x Genomics, cat. no. 2000095), 50 µl of 2× KAPA Hifi Hotstart ReadyMix (Kapa Biosystems, cat. no. 7958935001) and 40 µl of nuclease-free water. HTO libraries were purified with 1.2× SPRI bead selection. Size distribution and concentrations of cDNA and HTO libraries were verified on an Agilent Bioanalyzer High Sensitivity chip (Agilent Technologies, cat. no. 5067-4626). Finally, HTO and cDNA libraries were sequenced on a NovaSeq 6000 (Illumina) to obtain approximately 25,000 reads per cell.
3. Non-cell hashing experiment and 10x Genomics. Samples with a low number of cells in the previous experiment (samples of patient 365 and a subset of samples of patients 12 and 19) were analyzed using a non-cell hashing experiment (BCLLATLAS_29). Frozen samples were thawed and 1 ml of 37 °C pre-warmed Hibernate-E (Thermo Fisher Scientific, cat. no. A1247601) supplemented with 10% FBS (Thermo Fisher Scientific, cat. no 10082147) was added drop-wise with gently swirling of the sample. After 1 min of incubation at room temperature, 2,000 µl of pre-warmed medium was added as mentioned before. Samples were again kept at room temperature for 1 min and 5,000 µl pre-warmed medium was gently added. This step was conducted twice. Afterwards, samples were centrifuged at 500*g* for 5 min. Supernatant was removed and pellets were resuspended in 500 µl 1× PBS supplemented with 0.05% BSA and stained with 4,6-diamidino-2-phenylindole (DAPI) (Thermo Fisher Scientific, cat. no. D1306) at 1 µM final concentration. DAPI-negative live individual cells were sorted with a BD FACSAria Fusion Flow cytometer (BD Biosciences) in 1× PBS supplemented with 0.05% BSA. After FACS, cells were partitioned into Gel Bead In Emulsions by using the Chromium Controller system (10x Genomics, cat. no. 1000204) aiming at a Target Cell Recovery of 5,000 total cells. Sequencing libraries were prepared using the v.3.1 single-cell 3′ mRNA kit (10x Genomics). After GEM-RT cleanup, cDNAs were amplified during 14 cycles. cDNA quality control and quantification were performed on an Agilent Bioanalyzer High Sensitivity chip (Agilent Technologies). Libraries were indexed by PCR using the Chromiumi7 Sample Index Plate (10x Genomics, cat. no. 220103). Size distribution and concentration were verified on an Agilent Bioanalyzer High Sensitivity chip (Agilent Technologies, cat. no. 5067-4626). Finally, libraries were sequenced on a NovaSeq 6000 sequencer aiming for 40,000 reads per cell.

#### Read alignment

Raw reads were aligned to the GRCh38 human genome with Cell Ranger (v.4.0.0), with the ‘chemistry’ parameter set to ‘SC3Pv3’ and the ‘expect-cells’ parameter set to 20,000 and 5,000 for cell-hashed and non-hashed libraries, respectively. The remaining parameters for cell-hashed libraries were specified as described in the ‘Feature Barcode Analysis’ pipeline of Cell Ranger. For Smart-seq2 libraries, alignment and quantification was performed using zUMIs (v.9.4e)^105^.

#### Demultiplexing of hashtag oligonucleotides

Expression matrices were imported into R (v.4.0.4) with the ‘Read10X’ function from Seurat (v.4.0.3)^106^. HTO counts were normalized with a centered log-ratio transformation applied across features. Each cell barcode was assigned to a specific time point of the disease with the function ‘HTODemux’ (positive.quantile = 0.99) of Seurat. Barcodes that were positive for two or more time points were labeled as doublets and discarded. Likewise, cell barcodes negative for all time points were excluded. Finally, Scrublet (v.0.2.1)^107^ was run to aid in the detection of doublets.

#### Quality control, normalization and dimensionality reduction

Cells that possessed <900 UMIs, <250 expressed genes or a mitochondrial expression >22.5% were considered as poor quality and removed. Similarly, genes expressed in three or fewer cells were filtered out. Following data normalization and correction (Seurat and NormalizeData), we performed PCA (Seurat, RunPCA) using the scaled expression (Seurat and ScaleData) of the top 2,000 highly variable genes (Seurat: FindVariableFeatures, selection.method = VST). For Smart-seq2 data, we filtered out cells with <150,000 counts, <550 expressed genes or mitochondrial expression >18%. Cells with more than 700,000 counts or 3,750 detected genes were excluded. Similarly, genes expressed in three or fewer cells were filtered out. To separate neoplastic cells from the microenvironment, we corrected the top 30 principal components (PCs) for sample-specific variation using Harmony (v.1.0)^108^, as implemented in the RunHarmony (group.by.vars = sample) function (SeuratWrappers package, v.0.3.0). Subsequently, these 30 corrected PCs were used to embed cells in a UMAP (Seurat, RunUMAP) and in a 20-nearest neighbors graph (Seurat, FindNeighbors) for visualization and clustering, respectively. Following Louvain clustering (Seurat, FindClusters, resolution = 0.1), we focused our downstream analyses only on tumor B cells (CD79A) due to the low number of microenvironment cells.

#### Dealing with confounders

We observed batch effects between 10x Genomics experiments. To avoid batch effects within samples of the same patient, we focused on the BCLLATLAS_10 experiment for patients 12, 19 and 3,299. Conversely, as we did not obtain a clear signal-to-noise separation in the HTO demultiplexing of case 365, we analyzed the cells obtained with BCLLATLAS_29. We also found some cell neighborhoods that harbored a high percentage of mitochondrial expression and a low number of detected genes. In such cases, we were more stringent with the thresholds or fetched and eliminated these clusters with FindClusters. We also excluded some clusters of doublets that expressed markers of microenvironment cells (erythroblasts, T cells or natural killer cells). Finally, for patient 3,299 in which one sample was obtained from peripheral blood (PB), whereas the others were obtained from bone marrow (BM), we focused solely on the BM samples to avoid misinterpretations. For patient 365, the CLL and RT time points were sampled from PB and lymph nodes, respectively. As the same RT sample profiled with bulk RNA-seq clustered with other RT samples from PB, we analyzed them jointly. After all the filtering, we recomputed the highly variable genes and PCAs. To avoid overcorrection, we used the top 20 PCs as input to RunUMAP and FindNeighbors, without rerunning Harmony.

#### Clustering and annotation

Louvain clustering was performed with the FindClusters function, adjusting the resolution parameter for each patient independently. To annotate each cluster, we ran a ‘one-versus-all’ DEA for each cluster (Seurat, FindAllMarkers, Wilcoxon rank-sum test), keeping only upregulated genes with a log_2_FC > 0.3 and a Bonferroni-adjusted *P* value <0.001. If markers were specific to a subset of the cluster, we further stratified it with the FindSubCluster function. On the contrary, if two clusters possessed similar markers, we merged them. The CellCycleScoring function was used to identify clusters of cycling cells.

#### DEA and GSEA

We conducted a DEA between RT and CLL clusters of each patient independently, merging cells from all time points (Seurat, FindMarkers, logfc.threshold = 0, only.pos = FALSE, Wilcoxon rank-sum test). To find finer-grained gene expression changes, only nonproliferative clusters were considered. Genes with a Bonferroni-adjusted *P* value <0.05 were considered as significant. The resulting list of genes (sorted by decreasing log_2_FC) was used as input to the ‘gseGO’ function of clusterProfiler (v.3.18.1, parameters: ont = ‘BP’, OrgDB = org.Hs.eg.db, keyType = ‘SYMBOL’, minGSSize = 10, maxGSSize = 250, seed = TRUE). We then removed redundancy in the output list of GO terms with the ‘simplify’ function (cutoff of 0.75) and filtered out GO terms with an adjusted *P* value <0.05. To convert the expression of specific GO terms of interest into a cell-specific score, we utilized the AddModuleScore function from Seurat.

#### CNA inference from scRNA-seq data

For each patient separately, we ran inferCNV (v.1.11.1) integrating all samples together. We used CLL cells as reference because (1) we aimed to identify CNAs acquired at RT and (2) CLL had flat copy number profiles in virtually all chromosomes according to WGS. CLL cells were downsampled to the number of RT cells. We initialized an ‘infercnv’ object (CreateInfercnvObject) using the raw expression counts and the gene-ordering file https://data.broadinstitute.org/Trinity/CTAT/cnv/gencode_v21_gen_pos.complete.txt. CNAs were predicted (infercnv, run, HMM = FALSE, denoise = FALSE) setting the cutoff parameter to 1 and 0.1 for Smart-seq2 and 10x data, respectively. We customized the plotting with the plot_cnv function.

#### Analysis of an external scRNA-seq dataset

We downloaded the expression matrices and metadata of the dataset from Penter et al.^43^ with the GEOquery (v.2.62.2) (Gene Expression Omnibus identifier GSE165087), created a single Seurat object with all cells from all samples and filtered poor-quality cells as specified in the original publication^43^. Dimensionality reduction, DEA, GSEA and gene signature scoring were performed as described above.

### Cellular respiration

Cryopreserved cells were resuspended on RPMI-1640 (Gibco, cat. no. 21875034) with 10% FBS (Gibco, cat. no. 10270-106) and 1% Glutamax (Gibco, cat. no. 35050-061) at a concentration of 3 million cells ml^−1^. After 1 h of incubation at 37 °C, cellular respiration was performed using O_2_k-respirometers (Oroboros Instruments). Two milliliters of cell suspension were added in each respirometer chamber. Cellular respiration was performed at 37 °C at a stirrer speed of 750 r.p.m. Respiratory control was studied by sequential determination of routine respiration (oxygen consumption in living cells resuspended on RPMI-1640 with 10% FBS and 1% Glutamax), oligomycin-inhibited leak respiration (2 µl ml^−1^, Sigma-Aldrich, cat. no. O4876, CAS, 1404-19-9), uncoupler-stimulated ETC measured by the sequential titration of the ionophore carbonyl cyanide m-chlorophenyl hydrazone (Sigma-Aldrich, cat. no. C2759, CAS, 555-60-2) and residual oxygen consumption after inhibition of the electron transfer system by the addition into the chamber of rotenone (0.5 µM, Sigma-Aldrich, cat. no. R8875, CAS, 83-79-4) and antimycin A (2.5 µM, Sigma-Aldrich, cat. no. A8674, CAS, 1397-94-0). Data acquisition and real-time analysis were performed using the software DatLab 7.4 (Oroboros Instruments). Automatic instrumental background corrections were applied for oxygen consumption by the polarographic oxygen sensor and oxygen diffusion into the chamber^109^. The same experimental workflow was used to study cellular respiration in CLL and RT cells after 1 h of treatment with IACS-010759 (Selleckchem, cat. no. S8731, CAS, 1570496-34-2) at 100 nM.

### Calcium flux analysis

Cryopreserved cells were resuspended on RPMI-1640 medium with 10% FBS, 1% Glutamax and 5% penicillin (10,000 IU ml^−1)^/streptomycin (10 mg ml^−1^) (Thermo Fisher, cat. no. S8731) at 10^6^ cells ml^−1^. After 6 h of incubation at 37 °C and 5% CO_2_, cells were centrifuged and resuspended on RPMI-1640 with 4 µM Indo-1 AM (Thermo Fisher, cat. no. I1223) and 0.08% Pluronic F-127 (Thermo Fisher, cat. no. P3000MP) for 30 min at 37 °C and 5% CO_2_. Cells were subsequently labeled for 20 min at room temperature with surface marker antibodies CD19 (Super Bright 600; Invitrogen, cat. no. 63-0198-42) and CD5 (PE-Cy5; BD Biosciences, cat. no. 555354) for the identification of tumoral cells (CD19^+^CD5^+^). Next, cells were resuspended on RPMI-1640 before flow cytometry acquisition. Basal calcium was measured during 1 min before stimulation, then cells were incubated during 2 min at 37 °C with or without 10 µg ml^−1^ anti-human F(ab′)2 IgM (Southern Biotech, cat. no. 2022-01) and 3.3 mM H_2_O_2_ (Sigma-Aldrich, cat. no. H1009). Finally, 2 µM 4-hydroxytamoxifen (4-OHT) (Sigma-Aldrich, cat. no. H6278) was added to all conditions before continue recording for up to 8 min. Intracellular Ca^2+^ release was measured on LSRFortessa (BD Biosciences) using BD FACSDiva software (v.8) by exciting with ultraviolet laser (355 nm) and appropriate filters: Indo-1 violet (450/50 nm) and Indo-1 blue (530/30 nm). Bound (Indo-1 violet) and unbound (Indo-1 blue) ratiometric was calculated with FlowJo software (v.10). Gating analysis was as follows: cell identification in FSC-A versus SSC-A plot, singlet identification in FSC-A versus FCS-H plot, tumoral cells (CD19^+^CD5^+^) in CD19 (Super Bright 600) versus CD5 (PE-Cy5) plot and Ca^2+^ release in time versus Indo-1 violet/Indo-1 blue plot using a kinetics tool. Optimized dilutions for the antibodies were 1:3 for CD19 and 1:10 for CD5.

### Cell growth assays

Cryopreserved cells were resuspended on PBS at a concentration of 10^7^ cells ml^−1^ and labeled with 0.5 µM CFSE Cell Tracer (Thermo Fisher, cat. no. C34554) for 10 min. Cells were centrifuged and resuspended on enriched RPMI-1640 medium with 1% Glutamax, 15% FBS, 1× insulin-transferrin-selenium (Merk, cat. no. I3146), 10 mM HEPES (Fisher Scientific, cat. no. BP299), 50 µM 2-mercaptoethanol (Gibco, cat. no. 21985-023), 1× Non-Essential Amino Acids (Gibco, cat. no. 11140-050), 1 mM sodium pyruvate (Gibco, cat. no. 11360-070) and 50 µg ml^−1^ gentamicin (Gibco, cat. no. 15710-064) at a concentration of 10^6^ cells ml^−1^ supplemented with 0.2 µM CpG DNA TLR9 ligand (ODN2006-TL9; InvivoGen, cat. no. TLRL-2006) and 15 ng ml^−1^ recombinant human IL-15 (R&D Systems, cat. no. 247-ILB-025)^110^. When indicated, cells were treated for 72 h with 100 nM IACS-010759. Cells were labeled for 20 min at room temperature with surface marker antibodies CD19 (Super Bright 600), CD5 (PE-Cy5) and annexin V (Life Technologies, cat. no. A35122) before acquisition in a LSRFortessa (BD Biosciences) using the BD FACSDiva software (v.8) and analyzed using FlowJo (v.10). Gating analysis for divided cells was as follows: cell identification in FSC-A versus SSC-A plot, singlet identification in FSC-A versus FCS-H plot, alive cells in annexin V (PacB) versus SSC-A plot, tumoral cells (CD19^+^CD5^+^) in CD19 (Super Bright 600) versus CD5 (PE-Cy5) plot and proliferating cells in the CFSE histogram. Optimized dilutions for the antibodies were 1:3 for CD19, 1:10 for CD5 and 1:3 for annexin V.

### Reporting summary

Further information on research design is available in the Nature Research Reporting Summary linked to this article.

## Online content

Any methods, additional references, Nature Research reporting summaries, source data, extended data, supplementary information, acknowledgements, peer review information; details of author contributions and competing interests; and statements of data and code availability are available at 10.1038/s41591-022-01927-8.

## Supplementary information

Supplementary Information List of Supplementary Tables and Supplementary Figures.

Reporting summary

Supplementary Tables Supplementary Tables 1–25.

## Data Availability

Sequencing data are available from the European Genome–phenome Archive (http://www.ebi.ac.uk/ega/) under accession no. EGAS00001006327. scRNA-seq expression matrices, Seurat objects and corresponding metadata are available at Zenodo (10.5281/zenodo.6631966).

## Code availability

R markdown notebooks used for mutational signature, bulk RNA-seq, H3K27ac and ATAC-seq analyses can be found at https://github.com/ferrannadeu/RichterTransformation. R markdown notebooks to reproduce the scRNA-seq analyses can be accessed at https://github.com/massonix/richter_transformation. Code to normalize DNA methylation data can be found at https://github.com/Duran-FerrerM/DNAmeth_arrays. Code to calculate the tumor cell content, CLL epitypes and epiCMIT from DNA methylation data can be found at https://github.com/Duran-FerrerM/Pan-B-cell-methylome.
