# Supplementary material for: Detection of early seeding of Richter transformation in chronic lymphocytic leukemia
Source: Nat Med. 2022 Aug 11;28(8):1662–71. doi: 10.1038/s41591-022-01927-8 (PMC9388377; doi:10.1038/s41591-022-01927-8)
Supplement: Supplementary file 1 — List of Supplementary Tables and Supplementary Figures. [file 41591_2022_1927_MOESM1_ESM.pdf]

nature portfolio

https://doi.org/10.1038/s41591-022-01927-8

Supplementary information

Detection of early seeding of Richter transformation in chronic lymphocytic leukemia

In the format provided by the authors and unedited

Supplementary Information

Detection of early seeding of Richter transformation in chronic lymphocytic leukemia

Nadeu, Royo, Massoni-Badosa, Playa-Albinyana, Garcia-Torre, et al.

Table of Contents

List of Supplementary Tables 2
Supplementary Figures 6

List of Supplementary Tables

1. **Supplementary Table 1:** Metadata and WGS/WES specifications
    a. Supplementary Table 1a: Metadata
    b. Supplementary Table 1b: WGS/WES specifications
    c. Supplementary Table 1c: Sex and age at CLL diagnosis
2. **Supplementary Table 2:** Immunoglobulin gene rearrangements and oncogenic translocations determined by IgCaller
3. **Supplementary Table 3:** Mutations (SNV and indels)
4. **Supplementary Table 4:** Copy number alterations
    a. Supplementary Table 4a: List of copy number alterations
    b. Supplementary Table 4b: Candidate driver genes affected by copy number alterations
5. **Supplementary Table 5:** Structural variants
6. **Supplementary Table 6:** DNA methylation analyses
    a. Supplementary Table 6a: Metadata for samples with DNA methylation data
    b. Supplementary Table 6b: Differentially methylated CpGs between CLL and RT
7. **Supplementary Table 7:** Bulk ChIP-seq of H3K27ac and transcription factor analysis
    a. Supplementary Table 7a: Samples and metadata
    b. Supplementary Table 7b: Number of changes
    c. Supplementary Table 7c: Richter-specific common changes
    d. Supplementary Table 7d: Annotated differential expression genes in Richter-specific common regions
    e. Supplementary Table 7e: Transcription factors (expressed in RT)
    f. Supplementary Table 7f: Transcription factors (differentially expressed between RT and CLL)
8. **Supplementary Table 8:** Bulk ATAC-seq analyses
    a. Supplementary Table 8a: Samples and metadata
    b. Supplementary Table 8b: Number of changes
    c. Supplementary Table 8c: Richter-specific common changes
    d. Supplementary Table 8d: Annotated differential expression genes in Richter-specific common regions
9. **Supplementary Table 9:** Coding mutations in CLL and RT
10. **Supplementary Table 10:** CLL and lymphoma driver genes according to previous literature
    a. Supplementary Table 10a: Driver gene list
    b. Supplementary Table 10b: Regions considered for driver genes
11. **Supplementary Table 11:** Bulk RNA-seq analyses
    a. Supplementary Table 11a: Samples and metadata
    b. Supplementary Table 11b: Differentially expressed genes between RT and CLL

   c. Supplementary Table 11c: GSEA using hallmark gene sets
   d. Supplementary Table 11d: GSEA using curated C2 canonical pathways
   e. Supplementary Table 11e: Gene Ontology (GO) analysis
12. **Supplementary Table 12:** SNV identified in 147 CLL samples from the ICGC cohort and in 27 CLL post-treatment samples
13. **Supplementary Table 13:** Extraction and assignment of genome-wide mutational signatures
    a. Supplementary Table 13a: Single base substitution signatures extracted by HDP
    b. Supplementary Table 13b: Assignment of signatures extracted by HDP
    c. Supplementary Table 13c: Single base substitution signatures extracted by SignatureAnalyzer
    d. Supplementary Table 13d: Assignment of signatures extracted by SignatureAnalyzer
    e. Supplementary Table 13e: Single base substitution signatures extracted by SigProfiler
    f. Supplementary Table 13f: Assignment of signatures extracted by SigProfiler
    g. Supplementary Table 13g: Single base substitution signatures extracted by sigfit
    h. Supplementary Table 13h: Assignment of signatures extracted by sigfit
    i. Supplementary Table 13i. Comparison of SBS-RT with known signatures
14. **Supplementary Table 14:** Extraction and assignment of mutational signatures leading to clustered mutations
    a. Supplementary Table 14a: Single base substitution signatures extracted by HDP
    b. Supplementary Table 14b: Assignment of signatures extracted by HDP
    c. Supplementary Table 14c: Single base substitution signatures extracted by SignatureAnalyzer
    d. Supplementary Table 14d: Assignment of signatures extracted by SignatureAnalyzer
    e. Supplementary Table 14e: Single base substitution signatures extracted by SigProfiler
    f. Supplementary Table 14f: Assignment of signatures extracted by SigProfiler
    g. Supplementary Table 14g: Single base substitution signatures extracted by sigfit
    h. Supplementary Table 14h: Assignment of signatures extracted by sigfit
15. **Supplementary Table 15:** Fitting of genome-wide mutational signatures
    a. Supplementary Table 15a: Fitting of mutational signatures per sample (CLL/RT cohort)
    b. Supplementary Table 15b: Fitting of mutational signatures per sample (147 cases from the ICGC-CLL cohort)
    c. Supplementary Table 15c: Fitting of mutational signatures per sample (27 CLL post-treatment)
    d. Supplementary Table 15d: Presence of SBS-melphalan in CLL/RT samples (mSigAct)
    e. Supplementary Table 15e: Fitting of mutational signatures per clone
16. **Supplementary Table 16:** Characterization of SBS-RT
    a. Supplementary Table 16a: SBS-RT in coding gene mutations
    b. Supplementary Table 16b: Activity of mutational processes on specific chromatin states (RT-private mutations)

   c. Supplementary Table 16c: Enrichment of SBS-RT on specific chromatin states (RT-specific mutations)
   d. Supplementary Table 16d: Activity of mutational processes on early/late replication regions (RT-private mutations)
   e. Supplementary Table 16e: Enrichment of SBS-RT on early-late replication (RT-private mutations)
   f. Supplementary Table 16f: Replication strand bias analysis in RT- private mutations
   g. Supplementary Table 16g: Transcriptional strand bias analysis in RT- private mutations
17. **Supplementary Table 17:** Subclonal reconstruction from WGS
   a. Supplementary Table 17a: MCMC sampler details and tolerated errors
   b. Supplementary Table 17b: Clusters identified
   c. Supplementary Table 17c: Abundance of clusters in each time point
18. **Supplementary Table 18:** High-coverage, UMI-based NGS analysis
   a. Supplementary Table 18a: Metadata
   b. Supplementary Table 18b: Targeted mutations
   c. Supplementary Table 18c: Design of the amplicon-based NGS panel
   d. Supplementary Table 18d: Results
19. **Supplementary Table 19:** Fitting of clustered mutational signatures
   a. Supplementary Table 19a: Kataegis identified in the ICGC-CLL cohort
   b. Supplementary Table 19b: Kataegis identified in the initial CLL (#1) and RT subclones
   c. Supplementary Table 19c: Fitting of mutational signatures in kataegis
20. **Supplementary Table 20:** Single-cell DNA-seq
   a. Supplementary Table 20a: Samples and metadata
   b. Supplementary Table 20b: Studied genes
   c. Supplementary Table 20c: Mutations identified by scDNA-seq (from Tapestri Insights)
   d. Supplementary Table 20d: Allele dropout and doublet rates
   e. Supplementary Table 20e: Count matrices (based on infSCITE)
21. **Supplementary Table 21:** Characterization of immunoglobulin heavy chain gene rearrangements using high-coverage NGS
   a. Supplementary Table 21a: Samples and summary (Lymphotrack, DNA-based)
   b. Supplementary Table 21b: IGH subclones identified in case 3495 at time point 1 (Lymphotrack)
   c. Supplementary Table 21c: IGH subclones identified in case 3495 at time point 2 (Lymphotrack)
   d. Supplementary Table 21d: IGH subclones identified in case 12 at time point 1 (Lymphotrack)
   e. Supplementary Table 21e: Samples and results (RNA-based)
22. **Supplementary Table 22:** Single-cell RNA-seq: metadata, QC, clusters, and marker genes
   a. Supplementary Table 22a: Samples and metadata
   b. Supplementary Table 22b: Marker genes for clusters of case 12
   c. Supplementary Table 22c: Marker genes for clusters of case 19

    d. Supplementary Table 22d: Marker genes for clusters of case 63
    e. Supplementary Table 22e: Marker genes for clusters of case 365
    f. Supplementary Table 22f: Marker genes for clusters of case 3299
    g. Supplementary Table 22g: Number of cells per time point and cluster
23. **Supplementary Table 23:** Single-cell RNA-seq: patient-specific DEA and GSEA
    a. Supplementary Table 23a: DEA for case 12 (RT vs CLL)
    b. Supplementary Table 23b: DEA for case 19 (RT vs CLL)
    c. Supplementary Table 23c: DEA for case 63 (RT vs CLL)
    d. Supplementary Table 23d: DEA for case 365 (RT vs CLL)
    e. Supplementary Table 23e: DEA for case 3299 (RT vs CLL)
    f. Supplementary Table 23f: GSEA for case 12 (RT vs CLL)
    g. Supplementary Table 23g: GSEA for case 19 (RT vs CLL)
    h. Supplementary Table 23h: GSEA for case 63 (RT vs CLL)
    i. Supplementary Table 23i: GSEA for case 365 (RT vs CLL)
    j. Supplementary Table 23j: GSEA for case 3299 (RT vs CLL)
24. **Supplementary Table 24:** Respirometry assays in intact CLL and RT cells
    a. Supplementary Table 24a: Samples and metadata
    b. Supplementary Table 24b: Measurements
    c. Supplementary Table 24c: Summary
25. **Supplementary Table 25:** BCR signaling and cell growth assays in CLL and RT cells
    a. Supplementary Table 25a: Samples and metadata
    b. Supplementary Table 25b: Mean fluorescent ratio Indo-1(violet)/Indo-1(blue)
    c. Supplementary Table 25c: Flow cytometry gating strategy and proliferation results

Supplementary Figures

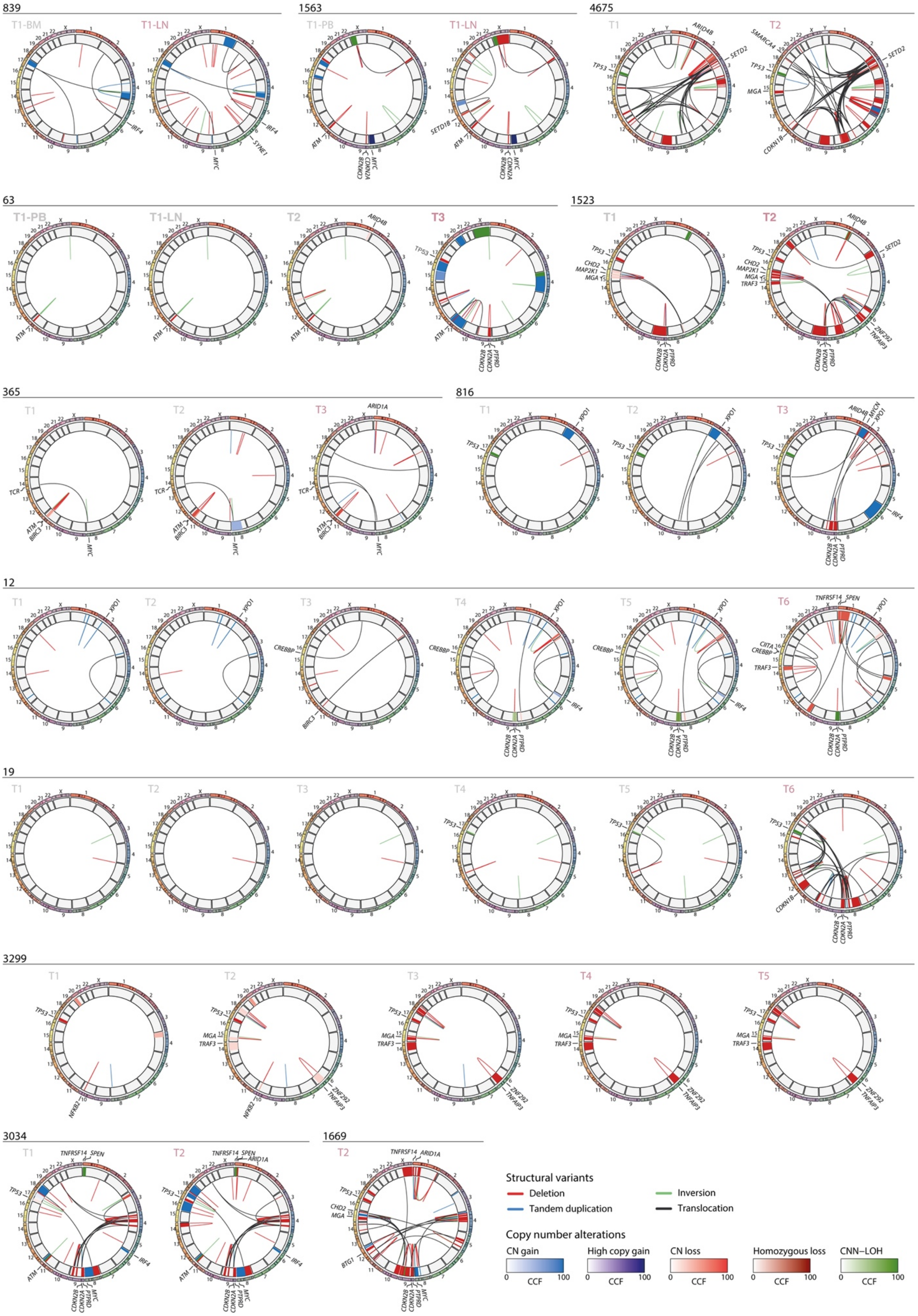

Supplementary Fig. 1. Structural alterations in CLL and RT. Circos plots illustrating the CNA and SV of each sample. Samples are grouped by patient. Chromosomes are displayed in the outer circle. The following section indicates the CNA painted according to their type. The transparency of the color is proportional to their cancer cell fraction (CCF). The inner circle represents the SV, linking together the breakpoints of the affected loci. Candidate driver genes affected by CNA and/or SV are annotated.

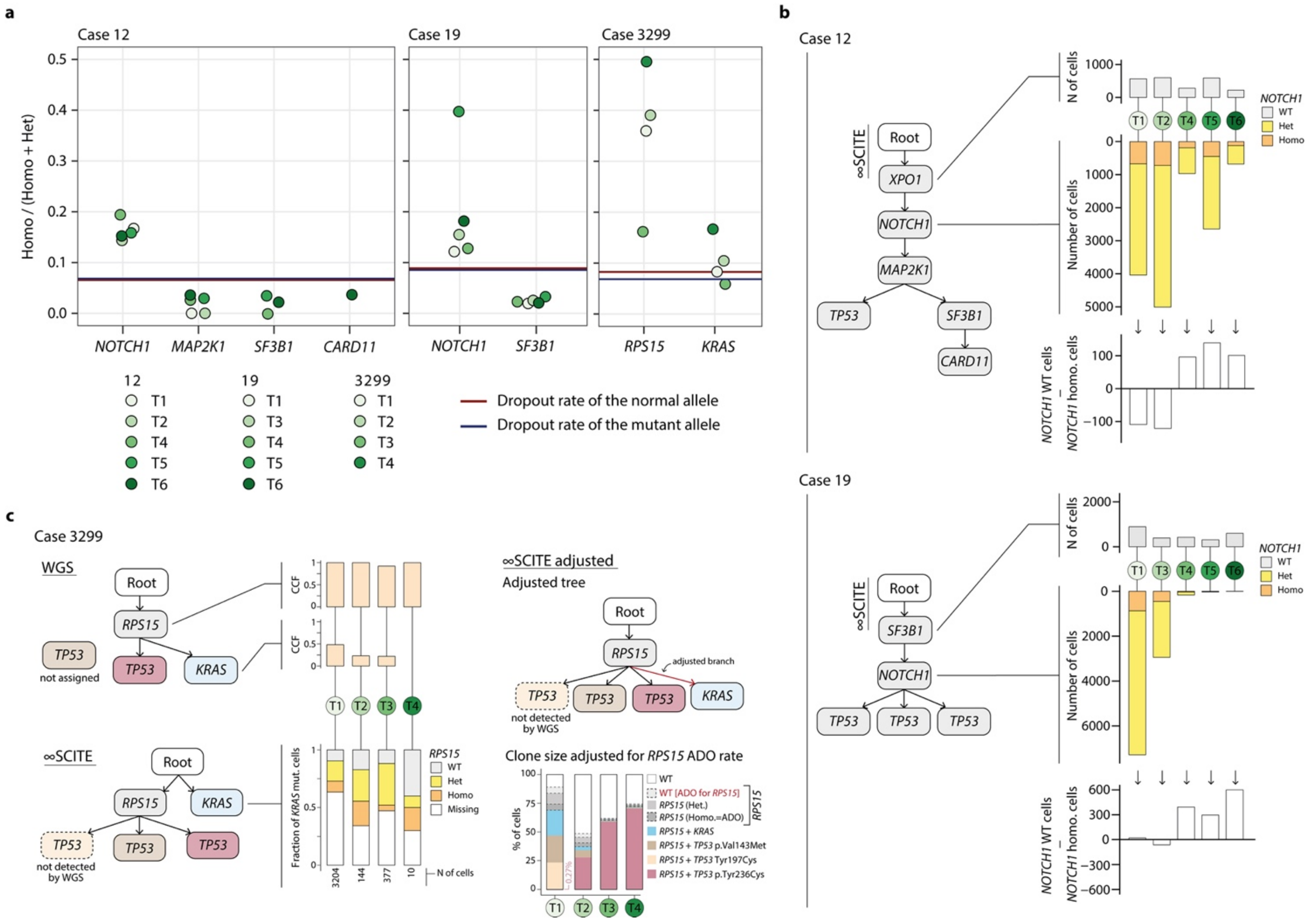

Supplementary Fig. 2. Case-specific considerations for scDNA-seq analyses. a. Allele dropout (ADO) rate of heterozygous mutations calculated based on the fraction of cells carrying a homozygous mutation (allele dropout of the wild-type allele). T, time point. Mean ADO rates calculated from heterozygous polymorphisms are represented with horizontal red/blue lines. *NOTCH1* and *RPS15* mutations had a higher ADO rate independently of the sample analyzed **b.** Mutation tree by ∞SCITE for case 12 and 19 when including the *NOTCH1* mutations [left]. For each case, the first bar plot on the right shows he number of cells carrying mutations only in *XPO1* or *SF3B1,* respectively, which are potential ADO of the *NOTCH1* mutated allele. The second bar plot shows the number of cells assigned to the *NOTCH1* node colored by the genotype of *NOTCH1*. Note that a remarkable fraction of these cells carried and homozygous *NOTCH1* mutations (ADO of the *NOTCH1* wild-type, WT, allele), which is similar to the *XPO1/SF3B1* only cells (potential ADO of the *NOTCH1* mutated allele) [bottom bar plot], which suggest that the obtained phylogeny in which the *NOTCH1* mutation is acquired after *XPO1* (case 12) or after *SF3B1* (case 19) could be influenced by its higher ADO rate. Consequently, we decided to show the results of the analyses excluding *NOTCH1* mutations to avoid a potentially artefactual order of *XPO1-NOTCH1* and *SF3B1-NOTCH1* in case 12 and 19, respectively. Nonetheless, the initial node of the mutation tree obtained without considering *NOTCH1* was labelled as carrying both mutations. **c.** Reconstructed mutation tree for case 3299 based on WGS [top left] and ∞SCITE [bottom left]. The first bar plot shows the cancer cell fraction (CCF) of *RPS15* and *KRAS* mutations by WGS, while the second one depicts the *RPS15* genotype of the cells assigned to the *KRAS* node by scDNA-seq.

Probably due to the high ADO rate of *RPS15*, ∞SCITE reported that *RPS15* and *KRAS* mutations were acquired in independent cells. However, in the first three time points analyzed, in which *KRAS* mutation was present in >10 cells, the fraction of *KRAS* mutant cells carrying a heterozygous or homozygous (ADO of the wild-type allele) *RPS15* mutation was higher than the number of *RPS15* wild-type cells. The high number of cells with missing *RPS15* genotype due to low sequencing coverage in this region could also impair the output of ∞SCITE. Therefore, we forced *KRAS* to be a branch of *RPS15* ('∞SCITE adjusted' plot). The bar plot on the bottom right side of the panel shows the procedure conducted to adjust for the high ADO rate of *RPS15*.

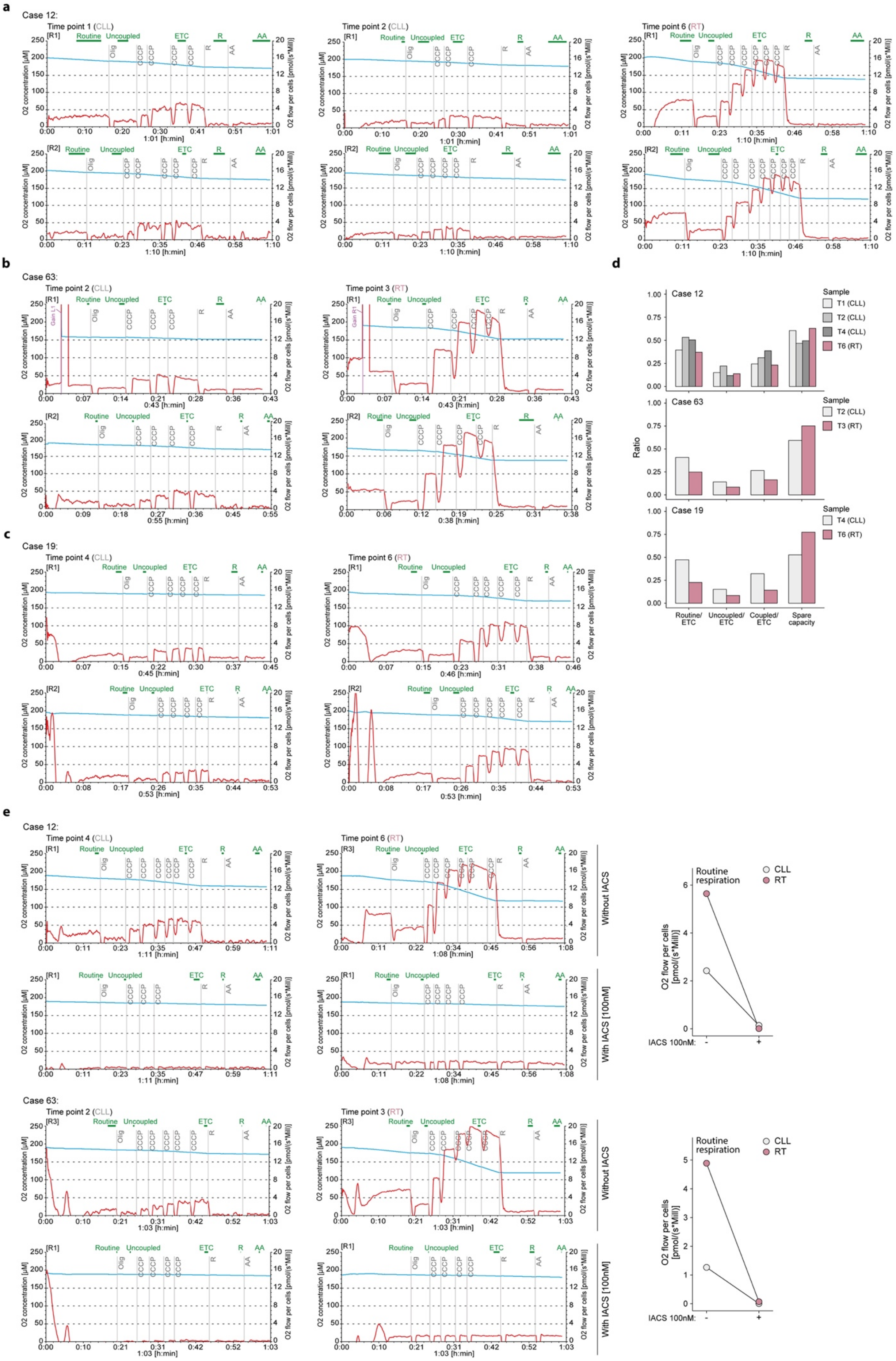
a
Case 12:
Time point 1 (CLL)
Time point 2 (CLL)
Time point 6 (RT)
Routine
Uncoupled
ETC
R
AA
Olig
CCCP
O2 concentration [µM]
O2 flow per cells [pmol/(s*Mill)]
1:01 [h:min]
1:10 [h:min]
b
Case 63:
Time point 2 (CLL)
Time point 3 (RT)
0:43 [h:min]
0:55 [h:min]
0:38 [h:min]
c
Case 19:
Time point 4 (CLL)
Time point 6 (RT)
0:45 [h:min]
0:46 [h:min]
0:53 [h:min]
d
Case 12
Case 63
Case 19
Sample
T1 (CLL)
T2 (CLL)
T4 (CLL)
T6 (RT)
T3 (RT)
Ratio
Routine/ETC
Uncoupled/ETC
Coupled/ETC
Spare capacity
e
Case 12:
Time point 4 (CLL)
Time point 6 (RT)
Without IACS
With IACS [100nM]
1:11 [h:min]
1:08 [h:min]
Case 63:
Time point 2 (CLL)
Time point 3 (RT)
1:03 [h:min]
Routine respiration
CLL
RT
O2 flow per cells [pmol/(s*Mill)]
IACS 100nM:

Supplementary Fig. 3. Respiratory capacity of intact CLL and RT cells. a-c. Overview of the respirometry assays conducted in intact CLL and RT cells of case 12 (a), 63 (b), and 19 (c). Two technical replicates of each sample [top and bottom] were performed. ETC, electron transfer system capacity; R, rotenone; AA, antimycin A; Olig, oligomycin; CCCP, ionophore carbonyl cyanide m-chlorophenyl hydrazine. **d.** Flux control ratios for case 12 [top], 63 [middle], and 19 [bottom]. Routine/ETC, routine respiration out of ETC; Uncoupled/ETC, uncoupled oxygen consumption out of ETC; Coupled/ETC, coupled oxygen consumption out of ETC (i.e., used to drive phosphorylation of ADP to ATP); Spare capacity; reserve respiratory capacity. **e.** Respirometry assays conducted in intact CLL and RT cells of case 12 [top] and 63 [bottom] with or without treatment with IACS-010759 (IACS) at 100nM for 1 hour [left]. Summary of the routine respiration of CLL and RT cells with and without IACS-010759 treatment.

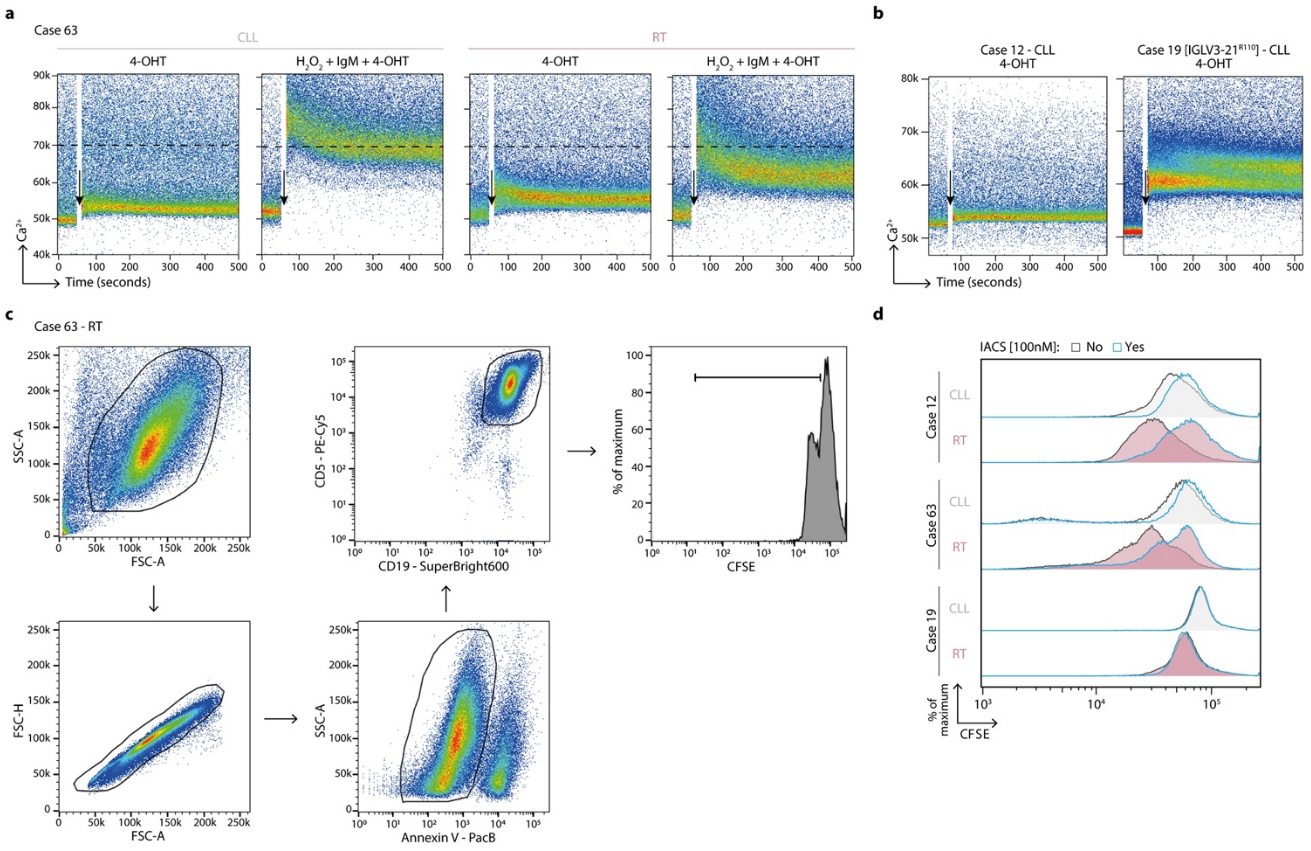

Supplementary Fig. 4. BCR signaling and proliferation experiments in CLL and RT cells. a. Ca^{2+} release kinetics by flow cytometry from case 63 in different timepoints (CLL and RT) with and without BCR stimuli (H_2O_2 + IgM). Different BCR activation was observed between CLL and RT samples with a lower Ca^{2+} release in RT. **b.** Ca^{2+} release kinetics by flow cytometry for CLL cells lacking the IGLV3-21^{R110} mutation (case 12) [left] or carrying the IGLV3-21^{R110} mutation (case 19) [right] upon incubation with 4-OHT without IgM stimulation. CLL cells from case 19 showed an autonomous BCR signaling in line with the presence of the IGLV3-21^{R110} mutation. **c.** Flow cytometry gating strategy in case 63 for proliferating cells. Gating analysis was as follow: cell identification in FSC-A vs. SSC-A plot, singlet identification in FSC-A vs. FCS-H plot, alive cells in AnnexinV - PacB vs. SSC-A plot, tumoral cells (CD19^{+} CD5^{+}) in CD19 - SuperBright600 vs. CD5 - PE-Cy5 plot, and proliferating cells in CFSE histogram. **d.** CLL and RT cells from cases 12, 63, and 19 were treated with IACS-010759 to assess growth inhibition, measured as CFSE low cells (% proliferating cells). Treated (blue line) or non-treated (black line) cells with IACS-010759 (IACS) at 100nM for 72 hours are represented in histograms as CFSE intensity vs. normalized cell count.
