# Supplementary material for: Detection of early seeding of Richter transformation in chronic lymphocytic leukemia
Source: Nat Med. 2022 Aug 11;28(8):1662–71. doi: 10.1038/s41591-022-01927-8 (PMC9388377; doi:10.1038/s41591-022-01927-8)
Supplement: Supplementary file 2 — Reporting summary [file 41591_2022_1927_MOESM2_ESM.pdf]

nature portfolio

Corresponding author(s): Elías Campo, Ferran Nadeu

Last updated by author(s): Jun 22, 2022

Reporting Summary

Nature Portfolio wishes to improve the reproducibility of the work that we publish. This form provides structure for consistency and transparency in reporting. For further information on Nature Portfolio policies, see our Editorial Policies and the Editorial Policy Checklist.

Statistics

For all statistical analyses, confirm that the following items are present in the figure legend, table legend, main text, or Methods section.

n/a	Confirmed	
☐	☒	The exact sample size (*n*) for each experimental group/condition, given as a discrete number and unit of measurement
☐	☒	A statement on whether measurements were taken from distinct samples or whether the same sample was measured repeatedly
☐	☒	The statistical test(s) used AND whether they are one- or two-sided<br>*Only common tests should be described solely by name; describe more complex techniques in the Methods section.*
☐	☒	A description of all covariates tested
☐	☒	A description of any assumptions or corrections, such as tests of normality and adjustment for multiple comparisons
☐	☒	A full description of the statistical parameters including central tendency (e.g. means) or other basic estimates (e.g. regression coefficient) AND variation (e.g. standard deviation) or associated estimates of uncertainty (e.g. confidence intervals)
☐	☒	For null hypothesis testing, the test statistic (e.g. *F*, *t*, *r*) with confidence intervals, effect sizes, degrees of freedom and *P* value noted<br>*Give P values as exact values whenever suitable.*
☐	☒	For Bayesian analysis, information on the choice of priors and Markov chain Monte Carlo settings
☒	☐	For hierarchical and complex designs, identification of the appropriate level for tests and full reporting of outcomes
☐	☒	Estimates of effect sizes (e.g. Cohen's *d*, Pearson's *r*), indicating how they were calculated

Our web collection on statistics for biologists contains articles on many of the points above.

Software and code

Policy information about availability of computer code

Data collection	BD FACSDiva™ Software (v8.0), a collection of rich tools for flow cytometer and application setup, data acquisition, and data analysis that help streamline flow cytometry workflows. Expression matrices and metadata from GEO were downloaded with the R package GEOquery (v2.62.2). No software was used for the remaining data collection.
Data analysis	The following commercial and open source code/programs have been used: Infinicyt (v2.0), BWA-mem (v0.7.15), biobambam2 (v2.0.65), FastQC (v0.11.5), Picard tools (v2.10.2 and v2.8.1), Sidrón (Puente et al, Nature 2015), CaVEMan (cgpCaVEManWrapper, v1.12.0), Mutect2 (GATK v4.0.2.0 and v.4.0.4.0), MuSE (v1.0 rc), bcftools (v1.8 and v1.9), SMuFin (v0.9.4), Pindel (cgpPindel, v2.2.3), SvABA (v7.0.2), and Platypus (v0.8.1), somaticMutationDetector.py script (https://github.com/andyrimmer/Platypus/blob/master/extensions/Cancer/somaticMutationDetector.py), snpEff/snpSift (v4.3t), Battenberg (cgpBattenberg, v3.2.2), ASCAT (ascatNgs, v4.1.0), BRASS (v6.0.5), DELLY2 (v0.8.1), IgCaller (v1.2), alleleCounter (v4.0.0), Integrative Genomics Viewer (v2.9.2), VarScan2 (v2.4.3), VarDictJava (v1.4), LoFreq (v2.1.3.1), outLyzer (v1.0), and freebayes (v1.1.0), CNVkit (v0.9.3), Nexus 9.0 software (Biodiscovery), cutadapt (v1.15), fgbio (v1.3.0), ARResT/AssignSubsets online tool (http://tools.bat.infspire.org/arrest/assignsubsets/), IMGT/V-QUEST online tool (https://www.imgt.org/IMGT_vquest/input), trimmomatic (v0.36, v0.38), LymphoTrack MiSeq Data Analysis (v2.3.1, Invivoscribe Technologies), STARsolo (version STAR-2.7.9a), samtools (v1.10, v1.3.1), HDP (v0.1.5, https://github.com/nicolaroberts/hdp), SignatureAnalyzer (v0.0.7), SigProfiler (SigProfilerExtractor, v1.0.8), and sigfit (v2.0.0), MutationalPatterns R package (v3.0.1), mSigAct (v2.1.1), TxDb.Hsapiens.UCSC.hg19.knownGene R package (v3.2.2), minfi R package (v1.34.0), IlluminaHumanMethylationEPICanno.ilm10b4.hg19 R package (v0.6), BWA-ALN (v0.7.7), PhantomPeakQualTools (v1.1.0), MACS2 (v2.1.1.20160309), bedtools (v2.25.0), DESeq2 R package (v1.26.0 and 1.28.1), sva R package (v3.36.0), AME tool from MEME suite (https://meme-suite.org/meme/doc/ame.html), SortMeRNA (v4.3.2), kallisto (v0.46.1), tximport R package (v1.14.2), clusterProfiler R package (v3.14.3), Tapestri Pipeline (V1, Mission Bio), Genome Analysis Toolkit (GATK, v3.7), Tapestri Insights (v2.2, Mission Bio), ∞SCITE (https://github.com/cbg-ethz/infSCITE), Cell Ranger (v4.0.0), zUMIs (v.9.4e), Seurat R package (v4.0.3), Scrublet R package (v0.2.1), Harmony R package (v1.0), UpSetR R package (v1.4.0), GEOquery R package (v2.62.2), inferCNV (https://github.com/broadinstitute/inferCNV), DatLab 7.4 (Oroboros Instruments GmbH), BD FACSDiva software (v8), FlowJo software (v10).

March 2021

Custom code/tools: R markdown notebooks used for mutational signatures, bulk RNA-seq, H3K27ac, and ATAC-seq analyses can be found at https://github.com/ferrannadeu/RichterTransformation. R markdown notebooks to reproduce all scRNA-seq analyses can be accessed at https://github.com/massonix/richter_transformation. Code to normalize DNA methylation data can be found at https://github.com/Duran-FerrerM/DNAmeth_arrays. Code to calculate the tumor cell content, CLL epitypes, and epiCMIT from DNA methylation data can be found at https://github.com/Duran-FerrerM/Pan-B-cell-methylome.

For manuscripts utilizing custom algorithms or software that are central to the research but not yet described in published literature, software must be made available to editors and reviewers. We strongly encourage code deposition in a community repository (e.g. GitHub). See the Nature Portfolio guidelines for submitting code & software for further information.

Data

Policy information about availability of data

All manuscripts must include a data availability statement. This statement should provide the following information, where applicable:

- Accession codes, unique identifiers, or web links for publicly available datasets
- A description of any restrictions on data availability
- For clinical datasets or third party data, please ensure that the statement adheres to our policy

Sequencing data is available from the European Genome-phenome Archive (EGA, http://www.ebi.ac.uk/ega/) under accession number EGAS00001006327 (https://ega-archive.org/studies/EGAS00001006327). scRNA-seq expression matrices, Seurat objects, and corresponding metadata are available at Zenodo (https://doi.org/10.5281/zenodo.6631966).

Field-specific reporting

Please select the one below that is the best fit for your research. If you are not sure, read the appropriate sections before making your selection.

☒ Life sciences ☐ Behavioural & social sciences ☐ Ecological, evolutionary & environmental sciences

For a reference copy of the document with all sections, see nature.com/documents/nr-reporting-summary-flat.pdf

Life sciences study design

All studies must disclose on these points even when the disclosure is negative.

Sample size	No statistical methods were used for sample size determination. We included all patients with suitable material available. The analyzed cohort is representative of the main subtypes of the disease and cover the different treatment modalities.
Data exclusions	Data exclusion was performed for methylation and scRNA-seq data as detailed in the respective methods section. Briefly, for methylation analyses, 6 samples were removed due to a tumor cell content <60%, which impairs a proper methylation analysis. For scRNA-seq analyses, since BCLLATLAS_29 experiment did not cover all time points and several samples had poorer quality, we focused on the BCLLATLAS_10 experiment for cases 12, 19 and 3299. Conversely, as we did not obtain a clear signal-to-noise separation in the HTO demultiplexing of case 365, we analyzed the cells obtained with BCLLATLAS_29 for this case.
Replication	At least two experimental replication were conducted for the respirometry and cell growth assays with concordant results. The results of all replicates performed in these experiments are shown in the corresponding figures and tables. H&E and immunohistochemistry stainings were repeated twice with concordant results and an illustrative example of each is shown in Extended Data Figure 1. All other experiments/techniques were performed once.
Randomization	Not relevant to this study since no experimental groups and conditions were tested.
Blinding	Not relevant to this study since no group allocation nor outcome analyses were conducted.

Reporting for specific materials, systems and methods

We require information from authors about some types of materials, experimental systems and methods used in many studies. Here, indicate whether each material, system or method listed is relevant to your study. If you are not sure if a list item applies to your research, read the appropriate section before selecting a response.

Materials & experimental systems

n/a	Involved in the study
☐	☒ Antibodies
☒	☐ Eukaryotic cell lines
☒	☐ Palaeontology and archaeology
☒	☐ Animals and other organisms
☐	☒ Human research participants
☒	☐ Clinical data
☒	☐ Dual use research of concern

Methods

n/a	Involved in the study
☐	☒ ChIP-seq
☐	☒ Flow cytometry
☒	☐ MRI-based neuroimaging

Antibodies

Antibodies used

CD19 - SuperBright™ 600. Monoclonal anti-human mouse IgG1k; clone SJ25C1 from Invitrogen. Cat. no. 63-0198-42. Lot no. 2316679.
CD5 - PE-Cy™5. Monoclonal anti-human mouse IgG1k; clone UCHT2 (RUO) from BD Bioscience. Cat. no. 555354. Lot no. 1067970.

Validation

CD19 - SuperBright™ 600. RRID: AB_2637472.
CD5 - PE-Cy™5. RRID: AB_395758.

Human research participants

Policy information about studies involving human research participants

Population characteristics

The characteristics of the patients with chronic lymphocytic leukemia and Richter transformation included in this study are similar to previous clinical descriptions. Therefore, this cohort can be considered representative of this complication of the disease. The cohort includes patients in which the transformation occurred under different treatment conditions, as previously described: 1) At the moment of diagnosis without previous treatment; 2) After treatment with standard chemoimmunotherapy regimens; and 3) After one or multiple lines of treatment including novel target agents. Among the 19 patients studied, 10 were males and 9 females. Mean age at time of CLL diagnosis was 58.4 (range 38.9 to 78.2) years [individualized information can be found in Supplementary Table 1].

Recruitment

Patients included in the study were selected based on the following criteria: 1) diagnosis of chronic lymphocytic leukemia (CLL) which had evolved to prolymphocytic transformation, diffuse large B-cell lymphoma, or plasmablastic lymphoma (Richter transformation); 2) Availability of a cryopreserved sample of these Richter transformations that allowed extracting high-quality DNA for genomic studies. The samples were peripheral blood, bone marrow, or tissues, particularly lymph nodes, involved by the tumor; 3) Availability of a sample of their corresponding CLL phase of the disease and from a sample to obtain germ line DNA. 4) Clonal relationship of the RT and the CLL phase of the disease. The limitation of the study was that not all patients with CLL transforming to RT had suitable samples for genomic studies in the hospitals participating in the study. However, the clinical and pathological features of the selected patients were characteristic of the spectrum of features and alterations described in patients with Richter transformation. Some studies recognize as Richter transformation cases of diffuse large B-cell lymphoma not clonally related to the previous CLL. We excluded these cases because this situation represents a secondary tumor and not an evolution of the preceding CLL.

Ethics oversight

Written informed consent was obtained from all patients. The study was approved by the Hospital Clínic of Barcelona Ethics Committee.

Note that full information on the approval of the study protocol must also be provided in the manuscript.

ChIP-seq

Data deposition

☒ Confirm that both raw and final processed data have been deposited in a public database such as GEO.

☒ Confirm that you have deposited or provided access to graph files (e.g. BED files) for the called peaks.

Data access links
May remain private before publication.

Data is available from the European Genome-phenome Archive (EGA, http://www.ebi.ac.uk/ega/) under accession number EGAS00001006327 (https://ega-archive.org/studies/EGAS00001006327).

Files in database submission

All raw data (FASTQ files), processed and aligned data (BAM files), and called peaks are submitted to EGA.

Genome browser session
(e.g. UCSC)

No longer applicable.

Methodology

Replicates

No replicates were performed for the CLL and RT samples processed and analyzed for this project. Three biological replicates were performed for all mature B-cell subpopulations, as reported in the original publication (Beekman et al, Nature Medicine 2018).

Sequencing depth	Aimed average sequencing depth for ChIP-seq experiments was 60 million reads per sample (25 million uniquely mapped reads). New CLL/RT data was sequenced in single-end mode (1x50bp). B-cell data obtained from our previous publication (Beekman et al, Nature Medicine 2018) was sequenced at 2x50bp. The specific number of reads and number of uniquely mapped reads per sample are provided in the corresponding supplementary tables.
Antibodies	ChIP-seq of H3K27ac histone mark and ATAC-seq data were generated as described by the Blueprint consortium (http://www.blueprint-epigenome.eu/index.cfm?p=7BF8A4B6-F4FE-861A-2AD57A08D63D0B58). Catalog number of the antibody (Diagenode) used for H3K27ac is C15410196/pAb-196-050 (LOT: A1723-0041D).
Peak calling parameters	FASTQ files of ChIP-seq data were aligned to the reference genome (GRCh38) using BWA-ALN (v0.7.7, parameters: -q 5), duplicated reads were marked using Picard tools (v2.8.1, http://broadinstitute.github.io/picard), and low quality as well as duplicated reads were removed using samtools (v1.3.1, parameters: -b -F 4 -q 5 -b -F 1024). PhantomPeakQualTools (v1.1.0) were used to generate wiggle plots and for extracting the predominant insert-size as previously described (http://dcc.blueprint-epigenome.eu/#/md/methods). Peaks of H3K27ac were called using MACS2 (v2.1.1.20160309, parameters: -g hs -q 0.05 -keep-dup all -nomodel -extsize insert-size) as previously described (http://dcc.blueprint-epigenome.eu/#/md/methods). Peaks with q-values <1e-3 were included for downstream analyses. ATAC-seq FASTQ files were aligned to the reference genome (GRCh38) using BWA-ALN (v0.7.7, parameters: -q 5) and samtools (v1.3.1). BAM files were sorted and duplicates were marked using Picard tools (v2.8.1). Finally, low quality and duplicate reads were removed using samtools (v1.3.1, parameters: -b -F 4 -q 5 -b -F 1024). ATAC-seq peaks were determined using MACS2 (v2.1.1.20160309, parameters: -g hs -q 0.05 --keep-dup all -f BAM –nomodel –shift -96 –extsize 200) without input control. Peaks with q-values <1e-3 were included for downstream analysis.
Data quality	All assigned peaks had an FDR< 0.05. For H3K27ac, the mean number of peaks with a fold enrichment above 5 was 49% (median 54%). For ATAC-seq, the mean number of peaks with a fold enrichment above 5 was 82% (median 83%).
Software	In addition to the software mentioned before for read alignment and peak calling, we used bedtools (v2.25.e), DESeq2 and sva R package to process and analyze these data. Besides, custom R scripts have been used and are provided as R Markdown notebooks, which can be found at https://github.com/ferrannadeu/RichterTransformation.

Flow Cytometry

Plots

Confirm that:

☒ The axis labels state the marker and fluorochrome used (e.g. CD4-FITC).

☒ The axis scales are clearly visible. Include numbers along axes only for bottom left plot of group (a 'group' is an analysis of identical markers).

☒ All plots are contour plots with outliers or pseudocolor plots.

☒ A numerical value for number of cells or percentage (with statistics) is provided.

Methodology

Sample preparation	Cryopreserved cells from primary cells were thawed, counted, and resuspended in cell culture media. Cells were subsequently labeled for 20 minutes at room temperature with surface marker antibodies CD19 - SuperBright600 and CD5 - PE-Cy5 for the identification of tumoral cells (CD19+ CD5+). If cells were labeled also with AnnexinV - PacificBlue (cat. no. A35122; lot no. 2268389; Life Technologies), the incubation was done at 4ºC for 30 minutes. Next, cells were washed and resuspended up to 1x10^6 cells/mL prior acquisition. Additional details are provided in the Methods.
Instrument	BD LSRFortessa™ + HTS. Available laser lines: 405 nm (violet), 488 nm (blue), 561 nm (yellow/green) and 640 nm (red). Serial number: H17700035.<br>BD LSRFortessa™. Available laser lines: 355 nm (UV), 405 nm (violet), 488 nm (blue), 561 nm (yellow/green) and 640 nm (red). Serial number: H7J200001.
Software	BD FACSDiva™ v8.0 Software for data collection and FlowJo v10 Software for data analysis.
Cell population abundance	CD19 CD5 double positive cells, which identifies tumoral cells, were in an average >90% of the total population.
Gating strategy	Gating analysis was as follows: cell identification in FSC-A vs. SSC-A plot, singlet identification in FSC-A vs. FCS-H plot, tumoral cells (CD19+ CD5+) in CD19 - SuperBright600 vs. CD5 - PE-Cy5 plot and Ca2+ release in Time vs. Indo-1 violet / Indo-1 blue plot using kinetics tool.<br><br>Gating strategy for divided cells was as follows: cell identification in FSC-A vs. SSC-A plot, singlet identification in FSC-A vs. FCS-H plot, alive cells in AnnexinV - PacB vs. SSC-A plot, tumoral cells (CD19+ CD5+) in CD19 - SuperBright600 vs. CD5 - PE-Cy5 plot, and proliferating cells in CFSE histogram.<br><br>Additional details are provided in the Methods.

☒ Tick this box to confirm that a figure exemplifying the gating strategy is provided in the Supplementary Information.
